# Stabilizing Cu‐Based Photocathodes: From Interfacial Engineering to Advanced Architectures

**DOI:** 10.1002/gch2.202500555

**Published:** 2026-01-08

**Authors:** Alejandro García‐Eguizábal, Javier Llorente‐López, Laura Collado, Mariam Barawi, Marta Liras, Víctor A. de la Peña O'Shea, Miguel García‐Tecedor

**Affiliations:** ^1^ Photoactivated Processes Unit IMDEA Energy Móstoles Madrid Spain

## Abstract

Copper‐based oxides, including Cu_2_O, CuO, CuBi_2_O_4_, CuFeO_2_ and CuFe_2_O_4_ have emerged as promising photocathode materials for solar‐driven photoelectrochemical (PEC) reduction reactions such as hydrogen evolution (HER), carbon dioxide reduction (CO_2_RR), and nitrogen reduction (NRR). Their appeal lies in the combination of their earth‐abundance, low toxicity, and suitable optoelectronic properties. However, the practical deployment of these materials is hindered by their intrinsic instability under operating conditions, primarily due to photocorrosion, interfacial charge recombination, and limited carrier transport. This review provides a comprehensive overview of recent strategies developed to improve the stability of the most studied Cu‐based photocathodes in relevant reported works. Specifically, seven key approaches are discussed: (i) optimization of electrical contact with the substrate, (ii) use of hole‐selective layers, (iii) electron‐extraction overlayers, (iv) protective coatings, (v) surface passivation strategies, (vi) integration of co‐catalysts, and (vii) synergistic strategies. Particular emphasis is placed on how each strategy addresses specific degradation mechanisms, and how synergistic combinations can enable durable and efficient PEC operation. Finally, the present review outlines current challenges related to scalability, fabrication compatibility, and real‐world durability, and highlights emerging directions in materials design and device integration. Unlike previous reviews that predominantly compare device efficiencies, this work places stability at its core, providing a strategy‐oriented perspective on how Cu‐based photocathodes can be made durable under operational conditions. By systematically connecting structure, interface, and function, this work aims to guide the development of robust Cu‐based photocathodes for sustainable solar fuel production.

## Graphical abstract

This review examines the instability challenges of Cu‐based oxide photocathodes and presents several strategies that enhance their durability and performance in solar‐driven photoelectrochemical reactions. By linking structural, interfacial, and functional considerations, the article highlights how protective, selective, and synergistic approaches improve operational robustness and outlines future directions for scalable and practical solar‐fuel technologies.

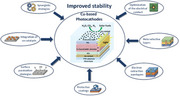

## 1 Introduction

The photoelectrochemical (PEC) route in artificial photosynthesis represents a promising and sustainable strategy to directly convert solar energy into chemical fuels. By mimicking the natural process of photosynthesis, it enables the transformation of abundant feedstocks such as water, carbon dioxide and nitrogen into hydrogen and added‐value carbon and nitrogen‐based products, offering an attractive alternative to fossil fuels and contributing to the transition toward a carbon‐neutral energy future [1]. In this scenario, copper‐based oxides have emerged as promising photocathode candidates for photoelectrochemical (PEC) reduction reactions [2, 3, 4]. Their appeal lies in the combination of earth‐abundance, low toxicity, and suitable optoelectronic properties, particularly their ability to absorb visible light due to relatively narrow band gaps ranging from ≈ 1.2 to 2.5 eV. Among them, cuprous oxide (Cu_2_O) is perhaps the most widely studied. It is a p‐type semiconductor with a direct bandgap of ≈ 2.0–2.2 eV, offering good visible light absorption and favorable conduction band alignment for proton reduction [5]. CuO, its fully oxidized counterpart, has a narrower bandgap (≈ 1.2–1.5 eV), which extends its light absorption into the near‐infrared, although it often suffers from lower charge mobility and complex surface chemistry [6]. In recent years, more complex copper‐based oxides such as CuBi_2_O_4_, CuFeO_2_, and CuFe_2_O_4_ have emerged as alternative photocathodes. CuBi_2_O_4_ combines visible light absorption with a relatively positive valence band, making it suitable for tandem systems [7], while CuFeO_2_, a delafossite oxide, offers a layered crystal structure and good photostability under certain conditions [8]. Finally, CuFe_2_O_4_, a spinel‐type oxide, exhibits strong light absorption and favorable charge separation due to its mixed‐valence structure, making it a promising candidate for photoelectrochemical reduction reactions [9].

These materials are particularly attractive for PEC applications involving not only HER, but also more complex transformations such as carbon dioxide reduction (CO_2_RR) and nitrogen reduction (NRR), and even urea production [10]. Their p‐type semiconducting behavior makes them suitable as photocathodes, facilitating photogenerated electron transfer to drive these thermodynamically demanding reactions. Additionally, the well‐known catalytic activity of copper toward C–C and C–N bond formation in electrocatalytic systems imparts additional functional versatility to PEC architectures [11, 12]. However, despite their potential, the practical deployment of Cu‐based oxide photocathodes is limited by poor operational stability, as a result of rapid photocorrosion and inefficient charge separation [13]. These limitations underscore the need for strategic material engineering through interface design, surface passivation, protective coatings and co‐catalyst integration to unlock their full capability in solar‐to‐chemical energy conversion.

Despite the large number of reports describing performance improvements in Cu‐based photocathodes, the field still lacks a dedicated and systematic analysis of the stabilization strategies that directly address their fundamental degradation pathways. In this review, we uniquely center the discussion on the chemical, structural, and interfacial routes developed to enhance the stability, rather than on device efficiency alone. Specifically, in this review, we summarize and analyze some of the most relevant advances in the stabilization of the most studied Cu‐based photocathodes, highlighting key approaches such as interfacial engineering, protective coatings, passivation strategies, and the integration of co‐catalysts. By organizing these strategies according to their functional roles, we aim to provide a clear framework for understanding how to improve both the performance and durability of these promising materials for solar‐driven reduction reactions.

## 2 Bare Cu‐Based Photocathodes

### 2.1 Copper (I) Oxide (Cu_2_O)

**FIGURE 1:**
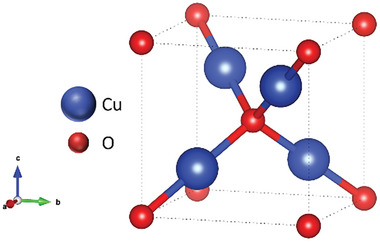
Simulated Cu_2_O cubic unit cell structure.

Among copper‐based oxides, cuprous oxide (Cu_2_O) stands out as one of the most extensively studied materials for photocathodic applications. Cu_2_O can be found in nature as cuprite mineral. Cu_2_O crystallizes in a cubic structure (*a* = *b* = *c* = 4.25 Å; *α* = *β* = *γ* = 90°), space group *Pn*3*m* (Figure 1), where oxygen atoms form a face‐centered cubic lattice and copper atoms occupy tetrahedral sites, a configuration that supports visible‐light absorption and charge transport in photoelectrochemical applications. With a direct bandgap of approximately 2.0 to 2.2 eV, Cu_2_O can efficiently absorb visible light, making it well suited for solar‐driven hydrogen evolution and other PEC reduction reactions [14]. Its conduction band position is sufficiently negative to drive proton reduction, while its p‐type semiconducting nature enables photogenerated electrons to migrate toward the electrolyte interface [15]. Moreover, Cu_2_O consists of earth‐abundant, low‐cost elements and can be synthesized using scalable and solution‐based methods, further reinforcing its potential for practical applications.

Despite these advantages, Cu_2_O suffers from significant challenges that limit its long‐term PEC performance. The material is prone to photocorrosion in aqueous environments, often undergoing self‐reduction to metallic copper or oxidation to CuO under illumination [16]. Moreover, it exhibits relatively short minority carrier diffusion lengths and high surface recombination rates, which hinder charge separation and extraction. As a result, bare Cu_2_O photocathodes typically display rapid degradation and poor stability under continuous operation [17].

### 2.2 Copper (II) Oxide (CuO)

**FIGURE 2:**
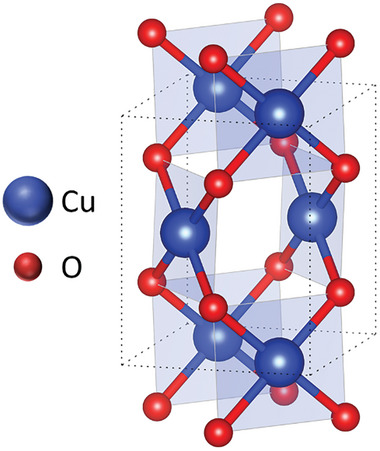
Simulated CuO monoclinic unit cell structure.

Copper(II) oxide (CuO), the fully oxidized form of copper, is another p‐type semiconductor that has garnered interest for photoelectrochemical (PEC) reduction reactions. CuO can be found in nature as tenorite mineral, also called black copper. CuO crystallizes in a monoclinic structure (*a* = 4.25 Å; *b* = 4.06 Å; *c* = 5.16 Å; *α* = 90°; *β* = 92.5°; *γ* = 90°), space group *C*2/*c* (Figure 2), where copper atoms are coordinated in a distorted square planar geometry, enabling narrow bandgap absorption and facilitating charge separation for photoelectrochemical reduction reactions [18]. With a narrow indirect bandgap in the range of 1.2 to 1.5 eV, CuO can harvest a broader portion of the solar spectrum compared to Cu_2_O, potentially offering higher theoretical photocurrent densities [19]. Its conduction band edge lies below the reduction potential for hydrogen evolution, enabling thermodynamically favorable electron transfer for proton reduction. In addition, CuO is chemically simple, cost‐effective, and readily synthesizable via low‐temperature routes such as electrodeposition, hydrothermal processing, and thermal oxidation of copper substrates.

Despite its attractive optical properties, CuO faces several intrinsic limitations that restrict its PEC performance. Chief among these are its poor charge transport properties, including low carrier mobility and short diffusion lengths, which contribute to high rates of bulk and interfacial recombination [20]. Furthermore, CuO exhibits significant chemical and photochemical instability under reducing conditions, often undergoing photocorrosion or reductive decomposition to metallic Cu, particularly under negative bias during operation [20]. This instability is further intensified by surface defects and the complex redox chemistry of copper species in aqueous environments.

### 2.3 Copper (II) Bismuth (III) Oxide (CuBi_2_O_4_)

**FIGURE 3:**
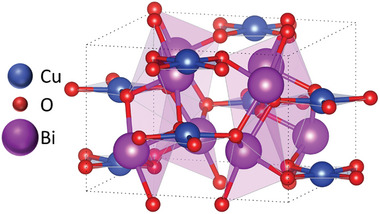
Simulated CuBi_2_O_4_ tetragonal unit cell structure.

CuBi_2_O_4_ is a complex copper‐based oxide that has emerged as a promising photocathode material for PEC applications, particularly in the visible light region. CuBi_2_O_4_ can be found in nature as kusachiite mineral. CuBi_2_O_4_ crystallizes in a tetragonal structure (*a* = *b* = 8.44 Å; *c* = 5.87 Å; *α* = *β* = *γ* = 90°) space group *P*4/*ncc* (Figure 3), featuring a layered arrangement of CuO_4_ square planes and BiO_6_ octahedra, which promotes visible‐light absorption and efficient charge separation [21]. It is a p‐type semiconductor with an indirect bandgap of approximately 1.5–1.8 eV, enabling effective absorption of visible light while maintaining a conduction band edge suitably positioned for driving reduction reactions such as hydrogen evolution [22]. The presence of bismuth introduces a layered crystal structure and contributes to a relatively positive valence band, which is advantageous for integration into tandem PEC devices where a high photovoltage is required from the photocathode side.

What makes CuBi_2_O_4_ particularly appealing is its chemical composition of abundant and non‐toxic elements, combined with synthetic flexibility through solution‐based methods or solid‐state reactions. However, its PEC performance is currently limited by several factors, including poor electrical conductivity, short hole diffusion lengths, and slow interfacial charge transfer kinetics [23]. In addition, like other copper oxides, CuBi_2_O_4_ is susceptible to photocorrosion and structural degradation under operating conditions, especially when exposed to reducing potentials in aqueous electrolytes.

### 2.4 Copper (I) Iron (III) Oxide (CuFeO_2_)

**FIGURE 4:**
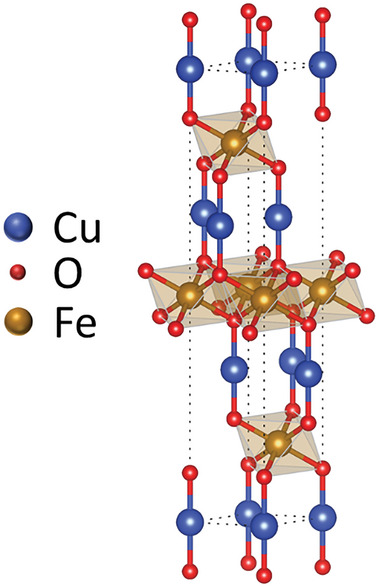
Simulated CuFeO_2_ rhombohedral unit cell structure.

CuFeO_2_ is a p‐type semiconductor that has gained attention as a photocathode material due to its structural stability and favorable electronic properties. CuFeO_2_ can be found in nature as delafossite mineral. CuFeO_2_ crystallizes in a trigonal delafossite structure (*a* = *b* = 2.88 Å; *c* = 16.91 Å; *α* = *β* = 90°; *γ* = 120°) space group *R*3̅*m* (Figure 4), where linearly coordinated Cu⁺ ions alternate with edge‐sharing FeO_6_ octahedra, providing a suitable bandgap and layered architecture that support visible‐light‐driven photoelectrochemical processes [24]. With a narrow bandgap of approximately 1.4 to 1.6 eV, CuFeO_2_ can absorb a broad range of the visible spectrum, making it a suitable candidate for solar‐driven PEC reduction reactions. Its layered crystal structure, featuring linear O–Cu–O bonding motifs, offers unique anisotropic transport characteristics and facilitates interfacial engineering [25]. In addition, the material is composed entirely of abundant, non‐toxic elements, which supports its potential for scalable and environmentally sustainable PEC technologies, including N_2_ reduction to ammonia [26].

Despite its promising light absorption and chemical robustness, the PEC performance of CuFeO_2_ remains limited by several intrinsic factors. These include low carrier mobility, short diffusion lengths, and suboptimal band alignment for efficient charge separation and electron transfer. Moreover, like other Cu(I)‐based materials, CuFeO_2_ is prone to photochemical instability, particularly under reducing conditions, where copper may be oxidized or reduced, leading to phase transformation or degradation [27]. Surface states and defect‐mediated recombination further constrain the efficiency of charge collection.

### 2.5 Copper (II) Iron (III) Oxide (CuFe_2_O_4_)

**FIGURE 5:**
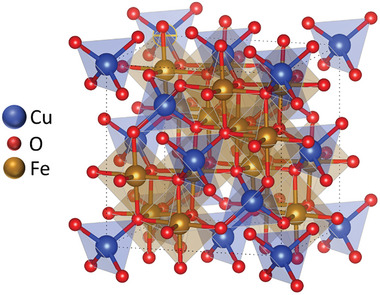
Simulated CuFe_2_O_4_ tetragonal unit cell structure.

Copper ferrite (CuFe_2_O_4_) is a spinel‐type p‐type semiconductor that has drawn increasing attention as a photocathode material for solar‐driven reduction reactions. CuFe_2_O_4_ can be found in nature as cuprospinel mineral. CuFe_2_O_4_ crystallizes in a tetragonal‐spinel structure (*a* = *b* = 5.80 Å; *c* = 8.73 Å; *α* = *β* = *γ* = 90°), space group *I*4_1_/*amd* (Figure 5), where Cu^2^⁺ and Fe^3^⁺ ions occupy both tetrahedral and octahedral sites within a close‐packed oxygen lattice, enabling strong light absorption and stable charge transport for photoelectrochemical reduction reactions [28]. With a bandgap typically ranging from 1.5 to 1.9 eV, CuFe_2_O_4_ can absorb a significant portion of the visible spectrum, making it a suitable candidate for PEC hydrogen evolution and other reduction processes [29]. Its conduction band edge is generally negative enough to support proton reduction, while the inclusion of iron expands its redox chemistry and introduces potential magnetic and catalytic functionalities. Moreover, CuFe_2_O_4_ is composed of earth‐abundant, low‐cost, and environmentally benign elements, which supports its appeal for scalable and sustainable energy applications.

Nevertheless, the PEC performance of CuFe_2_O_4_ has so far been modest, primarily due to low charge carrier mobility, severe bulk recombination, and sluggish surface reaction kinetics [30]. Additionally, CuFe_2_O_4_ suffers from limited photostability under operational conditions, where copper and iron ions may undergo redox transformations or leaching, leading to material degradation [30]. Surface defects and non‐ideal band alignment can also hinder effective charge separation and extraction at the semiconductor–electrolyte interface.

**FIGURE 6:**
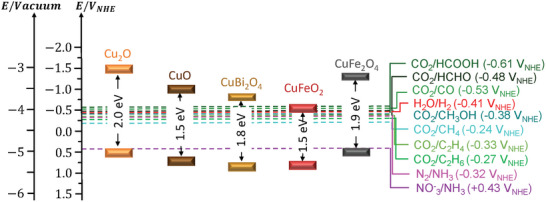
Band energy diagram at pH = 7 of the studied Cu‐based oxides with respect to the vacuum level and standard redox potentials for key solar‐driven reduction reactions.

Below, Figure 6 shows the band energy diagrams of the different Cu‐based photocathodes analyzed in this review, together with the redox potentials of the corresponding target reactions.

This diagram illustrates the conduction band minima (CBM) and valence band maxima (VBM) of the analyzed copper‐based oxides relative to the vacuum level and the normalized hydrogen electrodes (NHE). Overlaid on the diagram are the standard reduction potentials for the most important PEC reactions for solar fuels generation, including HER, carbon dioxide reduction to different products, and nitrogen and nitrate reduction to ammonia (NH_3_).

As it can be observed in Figure 6, the CBM positions of the different Cu‐based oxides indicate their thermodynamic ability to drive multielectron reduction reactions such as H_2_O to H_2_, CO_2_ to CO, CH_4_, C_2_H_4_, C_2_H_6_, etc., as well as N_2_ and NO_3_^−^ to NH_3_. The figure also presents the bandgap values of the analyzed Cu‐based oxides (ranging approximately from 1.5 to 2.0 eV), highlighting their ability for visible‐light absorption. The alignment shows that while most Cu‐based photocathodes can thermodynamically reduce protons and CO_2_‐derived species, their capacity to drive more demanding reductions (e.g., CH_4_, C_2_H_4_) depends on both the band edge position and interface engineering. Furthermore, although nitrate reduction is more favorable thermodynamically, achieving selectivity toward NH_3_ requires control over surface reaction pathways and catalyst properties.

This diagram underscores the importance of matching semiconductor band positions with redox reaction levels to enable efficient and selective PEC fuel production. To better understand the performance and limitations of copper‐based oxide photocathodes, Table 1 provides a comparative overview of their fundamental properties, stability, and behavior in PEC applications. Key parameters such as crystal structure, bandgap, light absorption range, semiconductor type, and intrinsic stability are highlighted, alongside practical aspects like synthesis feasibility and maturity of interfacial engineering strategies. The table also outlines their typical performance in HER, and notes their limited but emerging application in more demanding processes such as CO_2_RR and NRR.

**TABLE 1:** Comparison between different physical properties of the analyzed Cu‐based oxides.

Property	Cu_2_O	CuO	CuBi_2_O_4_	CuFeO_2_	CuFe_2_O_4_
Crystal structure	Cubic (cuprite)	Monoclinic	Tetragonal	Trigonal	Tetragonal
Cu oxidation state	Cu(I)	Cu(II)	Cu(II)	Cu(I)	Cu(II)
Bandgap (eV)	2.0–2.2	1.2–1.5	1.5–1.8	1.4–1.6	1.5–1.9
Semiconductor type	p‐type	p‐type	p‐type	p‐type	p‐type
Light absorption	Visible	Visible + NIR	Visible	Visible	Visible
Photocorrosion resistance	Low	Very low	Moderate	Moderate to good	Moderate
Carrier mobility / diffusion length	Moderate/short	Low/very short	Low/very short	Low	Low
Stability (bare electrode)	Poor	Very poor	Moderate	Moderate	Moderate
Synthesis simplicity	Easy	Very easy	Moderate	Challenging	Easy
Surface engineering maturity	High	Moderate	Moderate	Emerging	Moderate
Use in CO_2_RR / NRR	Moderate	Limited	Rare	Very rare	Very rare
Main challenges	Photocorrosion, surface recombination	Charge transport, instability	Poor conductivity, slow kinetics	Low mobility, difficult synthesis	High recombination, poor selectivity

Although each Cu‐based oxide exhibits distinct advantages and drawbacks, none of these materials are intrinsically stable in their unmodified form under operational conditions. This instability underscores the necessity of implementing stabilization strategies, including protective coatings, interfacial engineering, and co‐catalyst incorporation, which simultaneously promote charge transport and catalytic efficiency. The subsequent section outlines these strategies aimed at improving both stability and PEC performance.

It is important to highlight that the diverse Cu‐based oxides discussed in this review exhibit distinct intrinsic limitations, whether related to surface stability, interfacial recombination, or charge‐transport inefficiencies. These fundamental constraints provide the rationale for the various interfacial and structural engineering approaches compiled in the following section. The strategies summarized below should therefore be viewed as targeted responses to the general degradation pathways outlined previously, each addressing a different aspect of the broader stability and performance challenges inherent to Cu‐based photocathodes. Thus, the present review underscores that effective stabilization requires tailoring the chosen approach to the underlying material properties, while also recognizing that many of these strategies can be applied synergistically across different oxide systems.

## 3 Strategies for Enhancing the Stability of Cu‐Based Photocathodes

To overcome the intrinsic limitations of Cu‐based photocathodes (described above), a wide range of stabilization and performance‐enhancing strategies have been developed among the scientific community. These approaches aim to mitigate specific degradation pathways while simultaneously improving interfacial charge transport, selectivity, and catalytic activity. In the following sections, we categorize and discuss the most relevant strategies reported in the literature. Each strategy addresses a distinct set of challenges and, when combined synergistically, can significantly improve the durability and efficiency of Cu‐based photoelectrodes in photoelectrochemical applications.

To provide a clearer conceptual framework for the stabilization strategies discussed in this section, it is useful to relate them directly to the three dominant failure modes affecting Cu‐based photocathodes: photocorrosion, interfacial recombination, and poor carrier transport. Each of the approaches discussed below (optimization of electrical contact, use of hole‐selective layers, electron‐selective overlayers, protective coatings, surface passivation, co‐catalyst integration and synergistic strategies) targets one or more of these degradation pathways. In general, strategies involving contact optimization, hole and electron‐selective layers primarily mitigate interfacial recombination and enhance carrier extraction; protective coatings and passivation strategies are designed to suppress chemical degradation and photocorrosion; and co‐catalysts accelerate interfacial kinetics, thereby reducing both recombination losses and corrosion‐driven electron accumulation; synergistic strategies are a combination of them. By explicitly linking strategies to failure modes, the following section provides a structured view of how each approach contributes to the long‐term stability and performance of Cu‐based photocathodes.

### 3.1 Optimization of Electrical Contact With the Substrate

The electrical contact between a Cu‐based photocathode and its underlying conductive substrate (*e.g*., fluorine doped tin oxide (FTO), and Indium tin oxide (ITO)etc.) plays a critical role in determining the overall PEC performance of the device. Poor interfacial contact can lead to the formation of Schottky barriers, increased series resistance, and inefficient charge extraction, all of which significantly reduce the photocurrent and fill factor [31]. This issue, deeply studied in photovoltaic devices [32], is particularly pronounced in oxide semiconductors, where mismatches in work function and poor interfacial adhesion often hinder hole collection and facilitate charge recombination at the back contact [33, 34]. To address this challenge, one of the most effective approaches involves depositing ultrathin metallic interfacial layers between the Cu‐based absorber and the transparent conductive oxide (TCO) to form an ohmic or quasi‐ohmic contact, thereby facilitating hole transport and reducing interfacial energy barriers.

Among the different metals explored, gold has been frequently employed as metal back contact in Cu_2_O photocathodes [35, 36]. In the work of Paracchino et al. [37], the authors employed a double metallic layer deposition to improve the back contact in Cu_2_O photocathodes. Specifically, a 20 nm layer of Cr was deposited by thermal evaporation on the FTO substrate, followed by the thermal evaporation of 200 nm of Au. In the same direction, Dias and co‐workers studied the role of a 150 nm Au layer over FTO substrates in Cu_2_O photocathodes [38]. They observed the PEC response of the photocathodes suffered significantly in the absence of Au, with a very gradual onset and a *J*_ph_ reaching only −2 mA cm^−2^, compared with the −6 mA cm^−2^ at 0 V versus RHE when Au was present. This behavior indicates the presence of a significant resistive component within the device architecture. An essential function of the substrate/Cu_2_O interface is to establish an ohmic contact that facilitates efficient collection of photogenerated holes [39]. Given the relatively high work function of p‐type Cu_2_O, the choice of contact material is limited to those with similar or higher work functions. In this context, the interface between Cu_2_O and FTO does not form a truly ohmic junction, but a weak Schottky barrier, which hinders hole extraction and negatively impacts the *j*–*V* performance. Additionally, they observed that the electrodeposited Cu_2_O exhibits substrate‐dependent morphology, forming large, dispersed crystals on bare FTO, but yielding dense and uniform films on Au‐coated substrates. This difference influences both film quality and junction properties, highlighting the beneficial role of Au in device performance. They also proved how a very thin Au layer of 3 nm is enough to provide the same PEC response than the thick 150 nm, allowing also transparency and hence fully transparent configurations.

**FIGURE 7:**
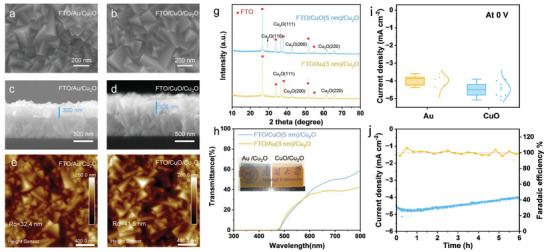
SEM top view and cross‐section images of a,c) FTO/Au/Cu_2_O and b,d) FTO/CuO/Cu_2_O. AFM images of e) FTO/Au/ Cu_2_O and f) FTO/CuO/Cu_2_O. g) X‐ray diffraction (XRD) patterns and h) transmittance spectra of Cu_2_O (50 min) on CuO (5 nm) and Au (3 nm). The inset presents a photograph of the Cu_2_O samples SEM images in Cu_2_O photocathodes with and without the presence of the Au layer showing their transparency. b) Photocurrent density at 0 V versus RHE of Cu_2_O electrodes on CuO (5 nm) and Au (3 nm) under AM 1.5G illumination. j) Stability test and Faradaic efficiency of FTO/CuO (5 nm)/ Cu_2_O (50 min) photocathode at 0 V versus RHE. The samples were tested in pH 5 buffered solution. Reproduced and adapted with permission from Wu et al. [40] Copyright 2025, Wiley.

On the other hand, Wu et al. [40] found that CuO can be used as a more efficient back contact that Au in Cu_2_O photocathodes. As explained above, Cu_2_O photocathodes commonly use gold as a back contact due to its high conductivity. However, beyond its limited optical transparency, gold is expensive and prone to facilitating electron–hole recombination at the Au/Cu_2_O interface. As shown in Figure 7, these authors demonstrated a simple and scalable approach to fabricate highly transparent Cu_2_O‐based devices by employing CuO, an earth‐abundant and more compatible alternative, as the back‐contact material.

Inefficient hole extraction in Cu‐based photocathodes has a direct and detrimental impact on their photoelectrochemical stability. Due to the p‐type nature of Cu‐based oxides, photogenerated holes must be efficiently transferred toward the back contact to prevent their accumulation near the surface. When this process is hindered, excess holes can oxidize the photocathode, leading to photocorrosion, the formation of insulating phases, and eventual structural degradation of the electrode. Moreover, hole accumulation enhances bulk and interfacial recombination, reducing photocurrent and device efficiency. In some cases, metallic layers can serve a dual function, acting as a diffusion barrier to prevent intermixing during processing or as a reflective layer to increase light absorption in thin‐film architectures. Consequently, strategies that facilitate rapid hole extraction are essential not only for boosting performance, but also for extending the operational lifetime of Cu‐based PEC systems.

Optimizing the electrical contact between the Cu‐based absorber and the substrate is essential for efficient charge extraction. However, achieving low‐resistance and stable contacts often relies on metallic interlayers or complex deposition techniques, which can increase fabrication costs and complicate scalability, particularly for large‐area or flexible devices.

### 3.2 Use of Hole‐Selective Layers (HSL) as Underlayers

Hole‐selective layers (HSLs) have emerged as an effective strategy to enhance both the performance and stability of Cu‐based photocathodes. These interfacial layers are typically inserted between the absorber and the conductive substrate to promote efficient hole extraction while simultaneously acting as a physical and chemical barrier that protects the copper interface from degradation. The effectiveness of an HSL depends largely on its valence band alignment with the Cu‐based material, as well as its ability to block electron back‐injection and suppress interfacial recombination.

**FIGURE 8:**
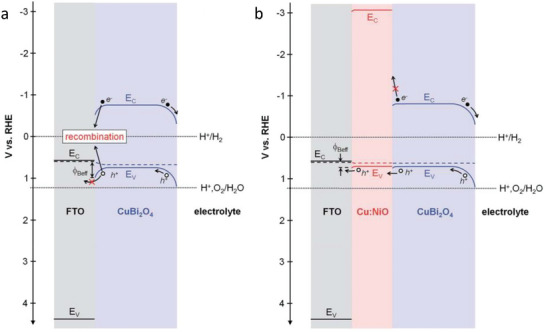
Energy band diagrams of a) FTO/CuBi_2_O_4_ photocathode and b) FTO/Cu:NiO/CuBi_2_O_4_ photocathode compared to electrochemical redox potentials for water reduction (H^+^/H_2_) and water oxidation (H^+^,O_2_/H_2_O). Reproduced and adapted with permission from Song et al. [41] Copyright 2019, The Royal society of Chemistry.

A variety of materials have been explored as HSLs for Cu‐based photocathodes. Transition metal oxides, such as NiO*_x_*, are particularly attractive due to their wide bandgaps, high transparency, and suitable work functions, which allow for favorable energetic alignment and directional charge transport. Son et al. [41] employed copper‐nickel mixed oxide (CuO/NiO), deposited by sequential sputtering deposition combined with an annealing process in air, as an efficient HSL in Cu_2_O photocathodes. The authors demonstrate, that compared with Au, CuO/NiO layer shows three significant improvements: i) it exhibits a better transparency than Au, ii) it is a hole selective layer that shows improved hole collection by blocking electron‐hole recombination and iii) it is a step towards low‐cost fabrication of Cu_2_O photocathodes. However, in terms of stability, both Cu_2_O and CuO/NiO are susceptible to be corrode when in contact with the electrolyte, and hence, the presence of protective layers (as TiO_2_) is still required. In a more recent work, Song et al. [42] reported the employment of Cu doped NiO as hole selective back contact in CuBi_2_O_4_ photocathodes. In this study, a very thin and transparent p‐type Cu doped NiO (Cu:NiO) back contact layer of 7 nm was deposited between the FTO substrate and CuBi_2_O_4_ by electron beam evaporation of Ni and Cu followed by post annealing in air. The introduction of Cu:NiO layer induced an increase of 25% in photocurrent density than photocathodes without the back contact layer. This improvement with the Cu:NiO back contact layer was attributed to hole selective transport across the CuBi_2_O_4_/Cu:NiO interface (Figure 8), which showed a smaller barrier height compared to the CuBi_2_O_4_/FTO interface.

In addition, Prévot et al. [43] employed CuAlO_2_ as an effective scaffold HSL to improve the PEC performance of CuFeO_2_ photocathodes. They found that CuAlO_2_ displayed favorable optoelectronic properties, being transparent in the visible range (*E*_g_ = 3.5 eV) and showing a flat band potential around 0.1 eV higher in energy than the one of CuFeO_2_. This band alignment promotes the selective extraction of photogenerated holes toward the substrate across the CuFeO_2_/CuAlO_2_ interface. The enhanced performance was attributed not only to improved charge separation, but also to the optimized morphology, specifically, a thin layer of CuFeO_2_ deposited over a highly porous CuAlO_2_ scaffold, which enabled greater incorporation of CuFeO_2_ into the electrode without exceeding the transport limitations of minority carriers.

**FIGURE 9:**
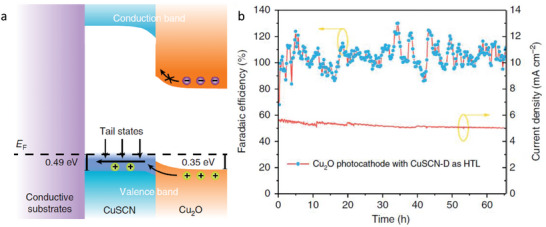
a) Band energy diagram of the Cu_2_O/CuSCN photocathodes showing the band‐tail assisted hole transport between. b) Chronoamperometry at 0.5 V versus RHE and corresponding Faradaic efficiency for HER in pH 5 buffered electrolyte. Reproduced and adapted with permission from Pan et al. [45] Copyright 2020, Springer Nature.

Another wide band semiconductor employed as HSL is CuSCN. This material has been widely used in optoelectronic devices due to its outstanding optical transparency, chemical stability, and efficient hole‐transport characteristics. In addition, its versatility in processing methods further enhances its appeal for the fabrication of electronic devices [44]. Specifically, CuSCN was reported as HSL in Cu_2_O photocathodes for water reduction in 2020 by Pan et al. [45] In this work two types of CuSCN with different structures were electrodeposited and compared. They found that the defective CuSCN showed a better performance that the more crystalline one. A detailed analysis of the optical, electrochemical, and electronic properties suggested that structural defects in CuSCN may actually enhance hole conduction. Furthermore, they found that hole transport between Cu_2_O and CuSCN is facilitated by valence band‐tail states (Figure 9a). Although the valence band offset between the two materials could, in principle, act as a barrier to hole transfer, the presence of these sub‐bandgap states enabled efficient hole transport without requiring direct transition through the valence band edge. By using CuSCN as HSL in Cu_2_O, the authors successfully reported stable PEC performance over 65 h of operation with a Faradaic Efficiency to hydrogen close to 100% (Figure 9b).

In addition, Tarek and co‐workers reported the use of CdSe as hole selective underlayer to improve the performance of CuFe_2_O_4_ in PEC CO_2_ reduction [46]. Structural and spectroscopic characterization (XRD, Raman, TEM, FESEM, EDX, XPS, UV–vis, and PL) confirmed the successful formation of the heterojunction. Compared to pristine CuFe_2_O_4_, the CdSe–CuFe_2_O_4_ photocathode exhibited markedly enhanced photoactivity, generating nearly six times higher photocurrent at 0.35 V versus NHE under CO_2_‐saturated conditions. The incident photon‐to‐current efficiency (IPCE) increased from 7.28% for CuFe_2_O_4_ to 12.09% for the heterostructure at 470 nm, evidencing more efficient visible‐light absorption and charge separation. Gas and liquid product analyses confirmed the selective formation of methanol with only trace H_2_ and CO byproducts. Notably, a methanol yield of 23.80 mmol L^−1^ cm^−2^ was achieved with Faradaic and quantum efficiencies of 72% and 16.9%, respectively, at 0.35 V bias. Mechanistically, the conduction band of CdSe acted as the active site for CO_2_ reduction by capturing photogenerated electrons, while water oxidation occurred at the valence band of CuFe_2_O_4_, enabling spatial charge separation and improved PEC performance. Photocurrent stability tests conducted at 0.35 V under continuous CO_2_ bubbling revealed excellent durability of the CdSe–CuFe_2_O_4_ photocathode. While both CuFe_2_O_4_ and the heterostructured composite showed stable responses, the CdSe–CuFe_2_O_4_ electrode exhibited virtually no photocurrent decay during prolonged operation. This negligible loss in current density indicates the non‐corrosive nature of the heterostructure under reductive conditions and highlights its strong potential for selective and sustained methanol production in PEC CO_2_ reduction systems. However, the use of CdSe as a hole‐selective layer introduces a significant trade‐off, as cadmium is a highly toxic and strictly regulated heavy metal. Although CdSe can provide excellent band alignment and improved charge extraction, its incorporation undermines one of the key benefits of Cu‐based systems, their potential for safe, scalable, and environmentally benign solar fuel production. For this reason, Cd‐based materials should be considered with caution, and future efforts may benefit from prioritizing cadmium‐free alternatives that maintain performance without compromising sustainability.

Choi and co‐workers recently shown the strategic use of an Sb–Cu_2_O buffer layer with a uniform (111) crystal orientation prior to the electrodeposition of Cu–Bi–O [47]. The buffer layer facilitated 2D film growth during electrodeposition while simultaneously supplying Cu throughout‐diffusion during annealing. This dual functionality enhanced the crystallinity of CuBi_2_O_4_, suppressed porous film formation, and produced a more uniform thin‐film structure with improved charge‐transport characteristics. Subsequent annealing promoted Cu diffusion from the Sb–Cu_2_O buffer into the CuBi_2_O_4_ layer, yielding dense CuBi_2_O_4_ (D‐CuBi_2_O_4_). Compared with conventional single‐layer CuBi_2_O_4_ (S‐CuBi_2_O_4_), the dense films exhibited superior onset potential, photocurrent density, and fill factor. The improved crystallinity and structural homogeneity facilitated more efficient charge separation, leading to significantly enhanced photoelectrochemical performance. The authors also observed that the S‐CuBi_2_O_4_ electrode displayed a rapid decline in photocurrent within the initial measurement period, reflecting its limited durability. In contrast, the D‐CuBi_2_O_4_ photocathode sustained a steady photocurrent with reproducible light on/off cycles, clearly evidencing its superior robustness under continuous operation. Then, the authors demonstrated how using Sb‐Cu_2_O buffer layer can noticeably boost the PEC performance of CuBi_2_O_4_ photocathodes.

Alternatively, the employment of organic HSLs have been also reported. The use of organic active layers always present important advantages in comparison with inorganic materials, such as their tunability, the employment of Earth‐abundant elements and their independence of geopolitical constrains. Specifically, PEDOT:PSS has been previously reported as an interlayer in a photocathode due to its noticeable potential to drive rapid charge separation and improve charge transfer kinetics [48]. In this context, recently, Ng et al. reported the employment of PEDOT:PSS as HSL in CuO photocathodes for PEC water splitting [49]. Due to its favorable energy band alignment, PEDOT:PSS enables efficient extraction and rapid transport of the photogenerated holes in the CuO absorber to the underlying FTO back contact.

Beyond energetic considerations, the interfacial morphology and chemical stability of the HSL layer are equally critical [50]. Achieving uniform, pinhole‐free deposition is essential to ensure complete surface coverage and to prevent electrolyte access to the underlying Cu‐based absorber. Moreover, the HSL must exhibit sufficient chemical robustness under PEC operating conditions to maintain its protective function over extended operation. By enabling selective hole transport and simultaneously shielding the sensitive copper interface, HSLs represent a multifunctional design element that significantly contributes to the longevity and efficiency of Cu‐based photocathodes. However, the deposition of HSLs can involve materials or methods that are not compatible with low‐cost or scalable processing. Careful selection of HSLs is necessary to balance performance with practical applicability.

### 3.3 Electron‐Extraction Overlayers

Electron‐extraction overlayers play a critical role in optimizing charge separation and collection in Cu‐based photocathodes by selectively transporting photogenerated electrons while suppressing interfacial recombination. To achieve this, the photoabsorber and ESL bands should obey to a staggered, type II band alignment scheme, that is, both the CB and VB of the ESL should lie below the corresponding CB and VB of the photoabsorber. Thus, the selective photogenerated electrons transport is attained while hole transport towards the ESL is hindered, suppressing interfacial recombination and improving photoelectrochemical properties of the device. These layers are typically deposited on top of the photoabsorber to improve band alignment, passivate surface defects, and facilitate directional charge transfer toward the electrolyte. Depending on the material system and device architecture, both organic and inorganic electron‐selective layers have been successfully integrated into Cu‐based PEC devices.

#### 3.3.1 Organic Overlayers

In hybrid or organic‐inorganic architectures, organic electron‐extraction layers such as conductive polymers (e.g., polyethylenimine, polythiophene derivatives) and fullerene‐based molecules (e.g., PCBM) have been explored due to their favorable electronic properties, tunable energy levels, and ease of solution processing. These materials can enhance electron selectivity and form conformal interfaces with rough or nanostructured absorber surfaces, enabling improved interfacial contact and reduced recombination. Moreover, their chemical versatility allows for taylor‐made properties through rational molecular design of their monomers and integration with soft or flexible substrates, opening pathways for novel device configurations.

Following this strategy, Karim and co‐workers developed a hybrid photocathode consisting on CuFe_2_O_4_ decorated with polyaniline (PANI), PANI@CuFe_2_O_4_, employed for PEC CO_2_ reduction in aqueous electrolyte [51]. The integration of polyaniline with CuFe_2_O_4_ significantly enhanced methanol production owing to two synergistic effects: (i) improved CO_2_ chemisorption on the photocathode surface and (ii) more efficient separation of photogenerated electron–hole pairs. As a result, the hybrid photocathode exhibited a methanol formation rate of 49.3 mmol g^−1^ h^−1^ with a Faradaic efficiency of 73%, while achieving incident photon‐to‐current efficiency (IPCE) and quantum efficiency (QE) values of 7.1% and 24.0%, respectively. This confirmed that coupling CuFe_2_O_4_, primarily serving as the light absorber, with PANI, acting as ESL and CO_2_ reduction site due to its favorable conduction band edge, provides a powerful platform for selective CO_2_‐to‐methanol conversion. Most importantly, stability tests revealed that PANI@CuFe_2_O_4_ could sustain continuous PEC operation for 4 h under visible light irradiation, maintaining performance and achieving a CO_2_‐to‐methanol conversion yield corresponding to a Faradaic efficiency of 35%.

Also, Kunturu and co‐workers presented a comprehensive approach to enhancing the efficiency and stability of Cu_2_O‐based photocathodes for photoelectrochemical (PEC) water splitting through controlled electrodeposition and carbon‐based surface protection. Cubic Cu_2_O films and porous Cu_2_O/CuO bilayer composites were synthesized under varying deposition and oxidation conditions to optimize morphological and electronic properties. The introduction of a carbon overlayer, applied via a simple solution‐based method, proved critical for achieving high stability and improved charge transfer efficiency. The optimized Cu_2_O/CuO/C photocathodes exhibited photocurrent densities of 6.5 and 7.5 mA cm^−2^ at 0 and −0.1 V versus RHE (pH 5.5), respectively, among the highest reported for copper oxide heterostructures in the absence of cocatalysts via simple synthetic methods and sustainable, abundant materials (sugar). Importantly, these photocathodes maintained their activity for over 50 h at a low applied bias (0.3 V vs RHE), demonstrating exceptional durability compared to uncoated Cu_2_O electrodes. This enhanced photostability was attributed to the protective and conductive role of the carbon coating, which minimized photocorrosion, suppressed dark currents linked to surface degradation, and facilitated efficient interfacial electron transport.

**FIGURE 10:**
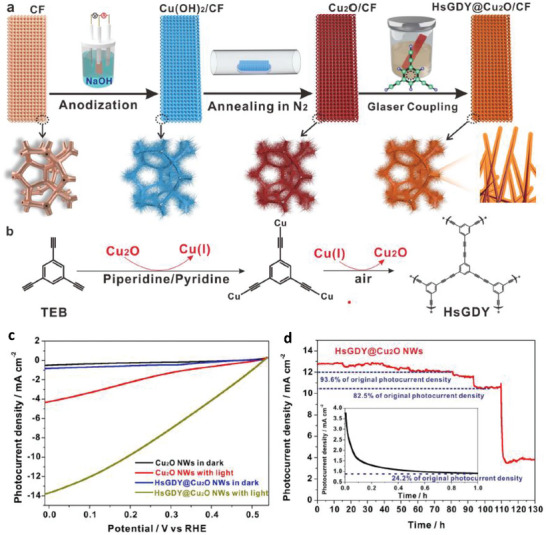
a) Schematic illustration of the fabrication of HsGDY@Cu_2_O/CF photocathode. b) Synthesis of HsGDY with Cu‐mediated Glaser coupling reaction. c) LSV plots of Cu_2_O NWs and HsGDY@Cu_2_O NWs in dark and under illumination of simulated 1 Sun solar light AM 1.5G, recorded with scan rate of 5 mV s^−1^ in 0.1 m Na_2_SO_4_ solution with pH = 4.9. c) Stability measurement of HsGDY@Cu_2_O NWs at potential of 0 V versus RHE, the inset is the chronoamperometric curve on Cu_2_O NWs. Reproduced and adapted with permission from Zhou et al. [52], Copyright 2022, Springer Nature.

In the same direction, Zhou et al. [52] introduced a novel in situ encapsulation strategy employing hydrogen‐substituted graphdiyne (HsGDY) to overcome the intrinsic instability and surface recombination limitations of Cu_2_O‐based photocathodes for photoelectrochemical (PEC) water splitting. By coating Cu_2_O nanowires (Cu_2_O NWs) with an ultrathin HsGDY layer, the researchers achieved both substantial improvements in charge separation and exceptional protection against self‐photocorrosion (Figure 10a). The resulting HsGDY@Cu_2_O NWs photocathode exhibited an outstanding photocurrent density of −12.88 mA cm^−2^ at 0 V versus RHE under standard solar illumination (Figure 10b), approaching the theoretical limit of Cu_2_O, and achieved a hydrogen evolution rate of 218.2 ± 11.3 µmol h^−1^ cm^−2^, further amplified under concentrated sunlight (10 suns) to 861.1 ± 24.8 µmol h^−1^ cm^−2^. Crucially, the long‐term PEC stability tests demonstrated the protective efficacy of the HsGDY layer. While unprotected Cu_2_O NWs rapidly degraded, retaining only 24.2% of their initial photocurrent after 1 h, the HsGDY‐encapsulated photocathodes maintained 93.6% of their initial activity after 80 h and 82.5% after 100 h of continuous illumination. Even after 120 h, the photocurrent remained stable at ≈3.5 mA cm^−2^, with minor degradation attributed to mechanical disruption of the encapsulation layer caused by vigorous hydrogen bubble formation (Figure 10c). Post‐reaction analyses revealed the partial emergence of metallic Cu, confirming localized exposure of the substrate where the protective coating was detached.

Deng et al. [53] reported the design of a Cu_2_O‐based photocathode coated with a metal–organic framework (MOF), Cu_3_(BTC)_2_, for efficient and durable photoelectrochemical (PEC) CO_2_ reduction. The in situ growth of the MOF layer on Cu_2_O provides a multifunctional interface that both protects the underlying semiconductor from photocorrosion and introduces catalytic sites for CO_2_ conversion. Ultrafast spectroscopy revealed that the Cu_2_O/MOF interface facilitates efficient charge separation and electron transfer, effectively suppressing recombination losses. As a result, the modified photocathode exhibits markedly improved activity and long‐term stability compared to bare Cu_2_O. The Cu_3_(BTC)_2_ layer fulfills three critical roles in PEC CO_2_ reduction: (i) it prevents photocorrosion and supports the controlled formation of trace metallic Cu species that enhance catalytic performance; (ii) it promotes interfacial charge transfer by acting as an electron relay between Cu_2_O and the reactant molecules; and (iii) it provides additional active sites for CO_2_ adsorption and reduction. Together, these effects lead to a substantial improvement in both efficiency and durability. The stability tests further underscore the protective function of the Cu_3_(BTC)_2_ coating. Chronoamperometric measurements under visible illumination revealed a nearly constant photocurrent density for the Cu_3_(BTC)_2_/Cu_2_O/ITO photocathode, in sharp contrast to the rapid photocurrent decay observed in the bare Cu_2_O electrode. Time‐dependent Faradaic efficiency and solar‐to‐chemical (STC) efficiency analyses confirmed the long‐term durability of the hybrid electrode during extended PEC operation. Post‐reaction characterizations (SEM, XPS, XRD, FT‐IR) showed that the MOF‐coated Cu_2_O maintained its structural and chemical integrity, exhibiting only slight electrochemical reduction, whereas the unprotected Cu_2_O underwent severe photocorrosion and oxidation to CuO, as evidenced by surface darkening and degraded performance. Interestingly, minor formation of Cu⁰ species within the MOF layer was detected after prolonged operation, which may contribute to the catalytic sites for CO_2_ reduction. However, excessive Cu⁰ formation at overly negative potentials was found to hinder catalytic efficiency.

Additionally, Zhang and co‐workers introduced a hybrid organic–inorganic photoelectrode design in which covalent triazine frameworks containing bithiophene moieties (CTF‐BTh) are conformally deposited onto Cu_2_O photocathodes and Mo‐doped BiVO_4_ photoanodes via electropolymerization [54]. The resulting thin CTF‐BTh films form favorable p–n and type‐II heterojunctions with the underlying metal oxides, optimizing band edge alignment and significantly enhancing both photoelectrochemical (PEC) performance and operational stability. The CTF‐BTh‐coated Cu_2_O photocathode demonstrated remarkable long‐term durability, maintaining ≈90% of its initial photocurrent after 150 h of continuous illumination at 0 V versus RHE, whereas the uncoated Cu_2_O electrode exhibited rapid degradation within just a few hours. Inductively coupled plasma mass spectrometry (ICP‐MS) revealed a stark difference in photocorrosion behavior: the Cu ion concentration in solution reached ≈4000 µg L^−1^ for bare Cu_2_O after only 4 h, compared to a negligible 0.3 µg L^−1^ for the protected electrode even after 150 h. This substantial improvement was attributed to the compact CTF‐BTh overlayer, which physically isolates the semiconductor from the electrolyte while facilitating efficient charge separation and transfer through the heterojunction. Furthermore, the same strategy applied to Mo‐doped BiVO_4_ yielded a stable photoanode, and when both modified electrodes were integrated into a tandem PEC cell, the unassisted solar water splitting device achieved a solar‐to‐hydrogen (STH) efficiency of 3.7%, retaining 3.24% after 120 h of continuous operation. This demonstrate the possibility of using Cu‐based photocathodes in PEC reactors at relevant operational conditions when protected from photocorrosion.

#### 3.3.2 Inorganic Overlayers

In contrast, inorganic overlayers such as TiO_2_, ZnO, SnO_2_, and CdS are widely used for their high electron mobility, optical transparency, and chemical stability under PEC operating conditions. These materials are often employed to tune band alignment, acting as electron‐selective contacts that facilitate charge transfer from the Cu‐based absorber to the electrolyte while blocking holes. Additionally, they serve as surface passivation layers, mitigating surface trap states that would otherwise lead to recombination losses. In some cases, these inorganic layers also contribute to physical protection against photocorrosion, especially when deposited via conformal techniques like atomic layer deposition (ALD).

Das and co‐workers reported the employment of an electron‐selective TiO_2_/graphene layer for inhibiting photocorrosion in Cu_2_O photocathodes [55]. To enhance durability under photoelectrochemical (PEC) operation, the authors introduced a dual protection strategy combining chemical vapor–deposited (CVD) graphene with an ultrathin amorphous TiO_2_ coating (≈10 nm). While the graphene interlayer effectively preserved the integrity of Cu_2_O during TiO_2_ deposition and provided substantial protection during PEC testing, the presence of microcracks allowed limited electrolyte penetration, resulting in gradual photocurrent decay. The subsequent deposition of amorphous TiO_2_ on top of the graphene successfully sealed these defects, providing complete protection to the Cu_2_O absorber and simultaneously functioning as an electron‐selective layer due to its favorable band alignment. As a result, the TiO_2_/graphene–protected Cu_2_O photocathode achieved a photocurrent density of −3 mA cm^−2^ at 0.0 V versus RHE under AM 1.5G illumination, approximately double that of the unprotected electrode (−1.4 mA cm^−2^), and exhibited markedly enhanced operational stability, maintaining a steady photocurrent beyond 600 s of light‐chopping chronoamperometry. Post‐test analyses revealed no evidence of Cu⁰ formation, confirming the suppression of Cu⁺/Cu⁰ photocorrosion pathways. The work underscores the effectiveness of hybrid graphene/TiO_2_ protection in stabilizing Cu_2_O photocathodes under neutral electrolyte conditions, while highlighting that further optimization of the TiO_2_ overlayer, particularly in mitigating Ti⁴⁺/Ti^3^⁺ reduction, is necessary to achieve long‐term electron selectivity and sustained PEC performance.

**FIGURE 11:**
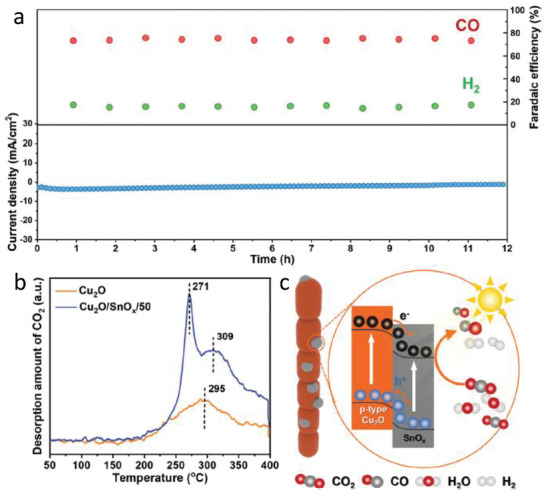
a) Plot of geometrical current density and Faradaic efficiencies for CO and H_2_ versus time over Cu_2_O/SnO_x_/50 hybrid NWs at a constant potential of −0.35 V versus RHE. b) CO_2_‐TPD profiles over Cu_2_O NWs and Cu_2_O/SnO_x_/50 hybrid NWs. c) The scheme of Cu_2_O/SnO_x_ heterojunction for PEC CO_2_ reduction. Reproduced and adapted with permission from Zhang et al. [56] Copyright 2022, Wiley.

Zhang et al. [56] studied a Cu_2_O/SnO_x_ hybrid nanowire (NW) photocathode capable of efficiently driving the photoelectrochemical (PEC) reduction of CO_2_ with H_2_O into syngas under visible light illumination. The hybrid photocathode achieved a total Faradaic efficiency of 90.3% at −0.35 V versus RHE along 12 h (Figure 11a), producing CO and H_2_ with tunable molar ratios ranging from 2.2:1 to 4.6:1. The introduction of a SnO_x_ overlayer onto Cu_2_O nanowires significantly improved the photoelectrocatalytic performance by enhancing CO_2_ adsorption capacity, promoting charge transfer, and accelerating intermediate formation during CO_2_ reduction (Figure 11b). Comprehensive electrochemical and spectroscopic analyses, including linear sweep voltammetry (LSV), incident photon‐to‐current efficiency (IPCE), electrochemical impedance spectroscopy (EIS), and temperature‐programmed desorption (TPD) confirmed the improved light harvesting and interfacial kinetics induced by the SnO_x_ modification. Moreover, in situ diffuse reflectance infrared Fourier transform spectroscopy (DRIFTS) revealed that visible‐light irradiation facilitated the accumulation and conversion of CO_2_ reduction intermediates, leading to efficient CO evolution. A key feature of this work is the remarkable operational stability of the Cu_2_O/SnO_x_/50 hybrid photocathode. During continuous PEC CO_2_ reduction over 12 h, the electrode maintained stable current density and consistent syngas composition, with Faradaic efficiencies of approximately 74% for CO and 16% for H_2_, closely matching their quasi‐steady‐state values. Post‐reaction characterization through SEM and XRD confirmed that the morphology and crystallographic integrity of the hybrid nanowires remained unchanged after prolonged operation, indicating that the SnO_x_ layer effectively suppressed photocorrosion and structural degradation (Figure 11c).

Altogether, these examples underscore the importance of incorporating electron‐extraction overlayers, whether organic or inorganic, for achieving higher photocurrents, improved stability, and better control over interfacial energetics in Cu‐based photocathodes. However, the implementation of protective layers requires careful balance between passivation and charge transport. If excessively thick or resistive, these coatings can impede carrier flow and lower photocurrent. Thus, optimizing their thickness, composition, and deposition technique is vital to preserve efficiency. However, some deposition techniques, especially atomic layer deposition (ALD), are expensive and challenging to scale, limiting their commercial viability. When properly designed, protective layers markedly extend the operational lifetime of Cu‐based photocathodes in PEC devices.

### 3.4 Protective Layers

Protective layers are a widely adopted strategy to enhance the operational stability of Cu‐based photocathodes by physically isolating the light‐absorbing material from the corrosive electrolyte environment. The analyzed copper oxides, particularly Cu_2_O and CuO, are highly susceptible to photocorrosion and electrochemical degradation under reductive PEC conditions. Protective coatings act as a barrier layer, preventing direct contact with water and reactive species, while still allowing photogenerated charge carriers to reach the interface where catalysis occurs.

Among the most commonly used protective materials are wide‐bandgap metal oxides deposited via atomic layer deposition (ALD), such as TiO_2_, Ga_2_O_3_, and Al_2_O_3_. These coatings are conformal, pinhole‐free, and offer excellent chemical robustness. TiO_2_, in particular, is widely favored due to its high transparency, good electron conductivity, and favorable band alignment with many Cu‐based semiconductors [57, 58].

**FIGURE 12:**
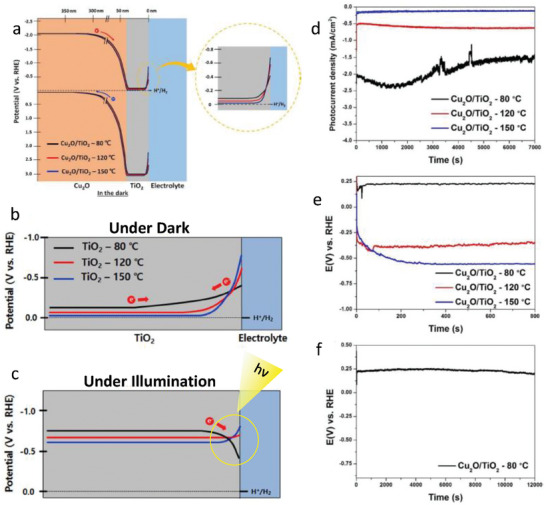
a) Band diagram for Cu_2_O/TiO_2_ photocathode with TiO_2_ grown at 80, 120, and 150°C biased at 0 V versus RHE in the dark. Schematic band diagrams of Cu_2_O/TiO_2_ ‐ 80, 120, and 150°C near the surface b) in the dark and c) under illumination at 0 V versus RHE. d) Chronoamperometric measurements at 0 V versus RHE for Cu_2_O/TiO_2_ ‐ 80, 120, and 150°C. e) Electrochemical potential required to maintain 1 mA cm^−2^ for Cu_2_O/TiO_2_ ‐ 80, 120, and 150°C. f) Cu_2_O/TiO_2_ ‐ 80°C exhibited good stability over 12000 s. Reproduced and adapted with permission from Choi et al. [59] Copyright 2016, Springer Nature.

Back in 2016, Choi et al. [59] reported a strategy to enhance the performance and durability of Cu_2_O photocathodes for PEC hydrogen production by engineering the band‐edge energetics of the protective TiO_2_ layer. They demonstrated that the deposition temperature of TiO_2_, grown via ALD, strongly influences its fixed charge density and, consequently, its interfacial electronic properties. When deposited at 80°C, the TiO_2_ layer provided energetically favorable conduction band alignment at the TiO_2_/electrolyte interface, enabling efficient charge transfer, suppressing electron accumulation, and preventing TiO_2_ reduction. This band‐edge‐engineered photocathode delivered a photocurrent density of −2.04 mA cm^−2^ at 0 V versus RHE under 1 sun illumination, representing a ≈1200% enhancement over the conventional 150°C‐deposited TiO_2_ layer, while also sustaining hydrogen evolution with improved Faradaic efficiency and stability for more than 2 h. Figure 12 shows the band diagram of the Cu_2_O/TiO_2_ photocathode (Figure 12a) and the TiO_2_/electrolyte interfaces under dark (Figure 12b) and under illumination conditions (Figure 12c). Figure 12d–f shows the stability measurements of the fabricated photocathode at different conditions. Beyond the specific Cu_2_O system, the study highlighted band‐edge control as a generalizable approach for designing stable and efficient protective layers, offering valuable insights for other unstable photoelectrodes such as Si, InP, and GaAs.

Al_2_O_3_ and Ga_2_O_3_, though often more insulating, can provide superior chemical passivation and are sometimes used in combination with conductive layers to achieve optimal protection. Also, the possibility of protecting a Cu‐based oxide photocathode with another Cu oxide has been reported. Lam and co‐workers shown the fabrication and optimization of a CuO/CuBi_2_O_4_ heterojunction photocathode deposited on an FTO substrate (FTO/CuO/CuBi_2_O_4_), designed to address the intrinsic photocorrosion and limited charge transport of CuO‐based electrodes in photoelectrochemical (PEC) water splitting [60]. The heterostructure was synthesized via a hydrothermal growth of CuO followed by successive spin coating of CuBi_2_O_4_ and thermal annealing, enabling precise control over morphology, composition, and interfacial quality. Among the tested samples, the photocathode comprising 15 CuBi_2_O_4_ coating layers annealed at 450°C exhibited the highest PEC performance, achieving a photocurrent density of 1.23 mA cm^−2^ at −0.6 V versus Ag/AgCl in neutral 0.1 m Na_2_SO_4_ electrolyte. Morphological analysis revealed a compact, crack‐free CuBi_2_O_4_ layer with continuous contact to the underlying CuO, which effectively facilitated charge carrier transport and suppressed interfacial recombination. The formation of a well‐defined CuO/CuBi_2_O_4_ heterojunction played a crucial role in enhancing both photoactivity and durability. CuO served as the primary light absorber and electron‐generating layer, while the CuBi_2_O_4_ overlayer acted as a protective and electron‐transporting medium. This outer layer effectively mitigated photocorrosion by preventing direct contact between CuO and the electrolyte, thereby suppressing self‐reduction processes (CuO → Cu_2_O/Cu⁰) and enabling more efficient charge separation. Furthermore, the heterojunction exhibited superior operational stability compared with bare CuO electrodes, maintaining steady photocurrent over extended illumination. The enhanced stability was attributed to the synergistic interaction between the two semiconductors, where CuBi_2_O_4_ not only provided chemical protection but also served as an efficient electron transport channel to the electrode/electrolyte interface, promoting the hydrogen evolution reaction.

On the other hand, Wi et al. [61] introduced a systematic approach to directly evaluate the influence of buffer and protection layers on charge separation and stability in Cu_2_O photocathodes. While Cu_2_O is one of the most efficient oxide‐based photocathodes for solar fuel production, its practical implementation is hindered by severe photocorrosion. Conventional strategies, such as integrating ZnO or TiO_2_ buffer layers, have been widely employed to suppress degradation and enhance charge extraction. However, their true ability to facilitate electron transfer has often been indirectly inferred from photocurrent measurements for hydrogen evolution, which are strongly affected by the presence of catalytic overlayers. To overcome this limitation, the authors employed 4‐hydroxy‐2,2,6,6‐tetramethylpiperidine‐1‐oxyl (TEMPOL) as a fast electron scavenger to decouple the effects of photocorrosion from intrinsic charge transport processes. Because the reduction rate of TEMPOL exceeds that of Cu_2_O self‐reduction, the measured photocurrent in its presence could be directly correlated with the efficiency of electron extraction to the interface. Using this approach, the study revealed that both electrodeposited ZnO and TiO_2_ layers effectively promoted electron transfer while simultaneously suppressing photocorrosion in Cu_2_O. Furthermore, the comparison between TiO_2_ films deposited by electrodeposition and atomic layer deposition (ALD) demonstrated that the deposition technique profoundly influences interfacial charge transfer behavior. Specifically, TiO_2_ layers fabricated via ALD failed to enhance the photocurrent relative to bare Cu_2_O, likely due to differences in defect states, conductivity, and interfacial atomic structure that govern electron transport at the Cu_2_O/TiO_2_ junction.

**FIGURE 13:**
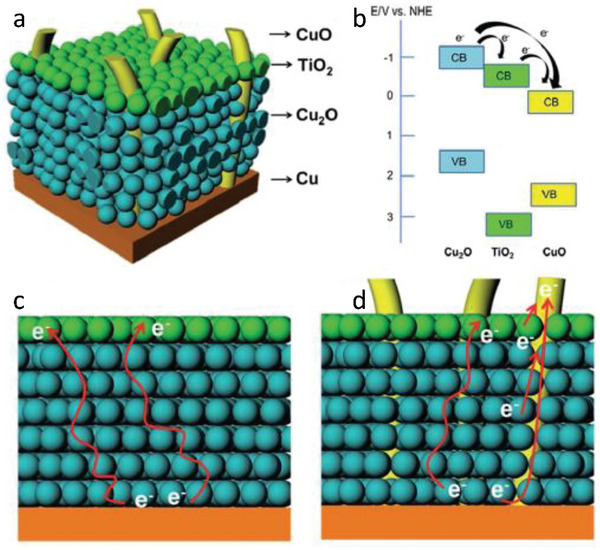
Schematic illustration of a) TiO_2_‐modified Cu_2_O–CuO photoelectrodes; b) corresponding band energy positions; electron diffuse transfer from Cu_2_O to interface c) without 1D CuO nanowires and d) with 1D CuO nanowires. Reproduced and adapted with permission from Wang et al. [62], Copyright 2015, RSC.

In 2015, Wang et al. [62] reported that introducing a TiO_2_ interlayer within Cu_2_O–CuO photocathodes markedly improves both photocurrent generation and photoelectrochemical stability under visible light. The TiO_2_ layer acted dually by suppressing detrimental interfacial electron conduction at the Cu_2_O/electrolyte boundary, thereby enhancing stability, while simultaneously promoting efficient electron transfer from Cu_2_O to CuO, which boosted the photocurrent (Figure 13). The optimized Cu_2_O–TiO_2_–CuO heterojunction thin film delivered a photocurrent of 2.4 mA cm^−2^ with 75% retention of activity, compared to only 1.3 mA cm^−2^ and 32% retention for unmodified Cu_2_O–CuO. Additionally, they found that the wavelength‐dependent IPCE confirmed enhanced charge collection in the Cu_2_O absorption region (400–560 nm), while transient fluorescence decay analysis provided evidence of accelerated electron transfer via TiO_2_. Structural engineering, including the embedding of 1D CuO nanowires beneath Cu_2_O, further reinforced stability and enabled direct electron transport pathways. Although complete suppression of photocorrosion was not achieved, they established TiO_2_ interlayers as an effective strategy for simultaneously passivating Cu_2_O surfaces and improving charge transport, opening pathways for further optimization of p‐type Cu_2_O photocathodes.

In 2021, Zhang and co‐workers reported a novel template‐assisted strategy was employed to prepare dendritic CuBi_2_O_4_ (CBO) photocathodes, which were subsequently stabilized with a conformal nanometer‐thick TiO_2_ passivation layer [63]. The dendritic architecture, derived from sacrificial Bi templates, enabled efficient light absorption, large electrode/electrolyte interfacial area, and shortened electron diffusion pathways, resulting in high photoactivity. The optimized CBO/TiO_2_ dendrites exhibited a photocurrent density of ≈0.90 mA cm^−2^ at 0.20 V versus RHE in neutral solution, which is among the highest reported values for CBO‐based photocathodes under comparable conditions. While bare CBO dendrites suffered from rapid photocurrent decay under continuous operation, the TiO_2_‐protected dendrites demonstrated markedly improved photostability. At 0.40 V versus RHE in neutral solution, the photocurrent of pristine CBO dropped sharply, whereas the CBO/TiO_2_ composite maintained a stable density of ≈0.39 mA cm^−2^ throughout the test. Similar results were observed in alkaline electrolyte (0.10 m NaOH), where bare CBO retained only 54.1% of its initial photocurrent after 6 h, but the CBO/TiO_2_ photocathode preserved 88.8% of its original value (≈0.36 mA cm^−2^) under identical conditions. Importantly, the absence of significant dark current confirmed the suppression of parasitic side reactions. This work highlights the dual role of the dendritic structure and TiO_2_ overlayer, where the former maximizes light harvesting and charge transport, and the latter effectively mitigates photocorrosion, ensuring operational durability well beyond previously reported CBO‐based systems.

In addition to pure oxides, hybrid protective coatings, including multilayer architectures or polymer‐inorganic composites, have been developed to tailor mechanical flexibility, adhesion, and ionic permeability. These advanced designs often aim to combine the chemical inertness of inorganic materials with the mechanical resilience of polymers or organosilicon compounds. Following the strategy of organic protective coatings, Zhang et al. [64] have recently reported a novel method to improve the PEC stability of Cu_2_O photocathodes by Ph─C≡C─Cu grafting. In this work, the authors developed a novel protective strategy to enhance the intrinsic instability of Cu_2_O photocathodes, which are otherwise prone to severe photocorrosion despite their high theoretical solar‐to‐hydrogen efficiency. They reported the self‐assembly of phenylethynyl copper (Ph–C≡C–Cu) layers on Cu_2_O surfaces via a photothermal method, forming a hydrophobic protective coating that effectively inhibited aqueous corrosion. The modified photocathodes retained up to 85% of their initial photocurrent density, compared to only 28% for bare Cu_2_O prepared on copper foam, and exhibited improved stability across various substrates, including Cu sheets and FTO. Although photocurrent densities were slightly reduced, the trade‐off was balanced by a substantial gain in durability. Characterization by SEM, XRD, and XPS confirmed the protective effect of the hydrophobic layer, consistent with increased water contact angles. The optimized Ph–C≡C–Cu/Cu_2_O/CF electrode enabled the construction of a two‐photoelectrode cell with BiVO_4_ photoanodes, achieving stable, bias‐free water splitting for more than 5 h with photocurrent densities of 1.26 mA cm^−2^. This study established Ph–C≡C–Cu as a promising metal–organic semiconductor coating to protect Cu_2_O against photocorrosion and extend its practical applicability in PEC devices.

These studies collectively highlight the importance of interfacial protection in sustaining photocathode performance. A critical consideration in the application of protective layers is also the trade‐off between protection and charge transport. Consequently, precise control over the layer's thickness, composition, and deposition method is essential to ensure that the protective function does not compromise PEC performance. Yet, many effective coatings, such as conformal oxides deposited via ALD, are costly and difficult to implement on large‐scale devices, and hence, balancing protection with manufacturability remains a key challenge. When properly engineered, such layers can significantly enhance the durability of Cu‐based photocathodes, enabling stable operation under realistic solar fuel generation conditions.

### 3.5 Surface Passivation Strategies

Surface passivation strategies are designed to mitigate interfacial charge recombination, one of the key loss mechanisms in PEC devices. In Cu‐based photocathodes, surface defects, such as vacancies, dangling bonds, or non‐stoichiometric terminations, can act as recombination centers that trap photogenerated carriers, significantly reducing photocurrent and photovoltage. Passivation layers serve to chemically neutralize or electronically saturate these defect sites, improving charge separation efficiency and enhancing device performance.

Unlike protective layers, which are typically physical barriers, passivation treatments often functionalize through chemical modification of the surface, without necessarily forming a continuous or impermeable coating. For instance, sulfurization treatments, commonly using Na_2_S or (NH_4_)_2_S, can replace oxygen‐terminated surface sites with sulfide species, reducing surface trap densities and improving electronic conductivity. Phosphonate or silane‐based molecular treatments have also been employed to form self‐assembled monolayers that passivate surface states while preserving access to the underlying semiconductor.

Several studies have demonstrated the effectiveness of these surface passivation strategies in enhancing the performance and stability of Cu‐based photocathodes. Xu and co‐workers fabricated of a thin porous inorganic–organic heterostructure photocathode, consisting of fluorine‐doped tin oxide (FTO) / CuBi_2_O_4_ (CBO) / polythiophene (PTh), for efficient and stable photoelectrochemical (PEC) water splitting [65]. The porous CuBi_2_O_4_ layer was optimized to enhance light absorption and charge transport, while surface passivation with the conducting polymer PTh was introduced to mitigate surface state trapping and suppress charge recombination. The resulting FTO/CBO/PTh electrode exhibited a photocurrent density of 0.41 mA cm^−2^ at 0.3 V versus RHE, approximately twice that of the pristine CBO electrode, attributed to the synergistic effects of improved light harvesting and more efficient charge separation at the heterojunction interface. The PEC stability of the heterostructured electrode was systematically evaluated under continuous 1‐sun illumination at a fixed potential of 0.4 V versus RHE. The FTO/CBO/PTh photocathode maintained nearly constant photocurrent for over 4000 s, showing a significant enhancement in durability compared to the uncoated FTO/CBO counterpart. Post‐reaction SEM analysis confirmed that the morphology and structural integrity remained unchanged after prolonged operation, demonstrating excellent chemical stability of the composite electrode. Importantly, these stability results were achieved without the use of sacrificial agents or noble metal cocatalysts, highlighting the cost‐effectiveness and practical relevance of this approach. The combination of a porous CuBi_2_O_4_ absorber and a PTh protective overlayer effectively balances light trapping, charge transport, and corrosion resistance.

Cao et al. [66] reported a simple yet effective surface modification strategy to enhance both the performance and stability of p‐type Cu_2_O photocathodes in photoelectrochemical (PEC) applications. In this work, the authors demonstrate that surface treatment of Cu_2_O with trisodium citrate (TSC) can significantly enhance PEC activity and durability without resorting to such intricate designs. The TSC‐functionalized photocathode (FTO/Au/Cu_2_O/TSC/TiO_2_/Pt) exhibited a twofold increase in photocurrent compared to untreated Cu_2_O, highlighting the effectiveness of this molecular modification. Mechanistically, TSC acts to stabilize surface Cu(II) species, increase doping density, and facilitate efficient charge carrier transfer to the electrolyte interface, thereby suppressing photocorrosion pathways.

**FIGURE 14:**
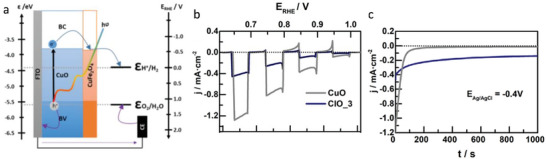
a) Band Alignment between CuO and CuFe_2_O_4_ in the core−shell structure of the nanowires. b) Comparison of the PEC performance of pristine CuO and CuO/CuFe_2_O_4_ photocathodes electrodes in N_2_‐purged 1 m NaOH; and c) chronoamperometric curves under illumination (320 mW cm^−2^, *λ* > 350 nm) at a potential of −0.4 V versus Ag/AgCl. Reproduced and adapted with permission from Cots et al. [67], Copyright 2018, ACS.

Cots and co‐workers presented the development of stable and efficient CuO‐based photocathodes through a simple and cost‐effective synthetic strategy [67]. The CuO films, composed of nanowires obtained by chemical oxidation of electrodeposited copper, initially deliver relatively high photocurrents in alkaline media (1 m NaOH). However, these photocurrents are partially attributed to reductive photocorrosion processes, as indicated by a faradaic efficiency for hydrogen evolution of approximately 45% in the absence of protection. To overcome this intrinsic instability, the authors introduced iron into the CuO nanowires via an impregnation and thermal treatment process, leading to the formation of a ternary copper–iron oxide (CuFe_2_O_4_) shell (Figure 14a). This surface phase transformation effectively stabilizes the photocathode, forming a diffuse CuO/CuFe_2_O_4_ passivating interphase that hinders interfacial charge recombination. Although this modification reduces the photocurrent to roughly one‐third of its original value (Figure 14b), it dramatically enhances the faradaic efficiency to nearly 100% for hydrogen evolution, even without the use of co‐catalysts. Chronoamperometric stability tests under continuous illumination (−0.4 V vs Ag/AgCl, 1000 s) confirm the protective role of the CuFe_2_O_4_ shell, with optimal performance achieved for a coating thickness of approximately 8 nm (Figure 14c). The favorable band alignment between CuO and CuFe_2_O_4_ facilitates efficient electron extraction while simultaneously preventing photocorrosion. Overall, this study highlights a straightforward and scalable route to stabilize CuO photocathodes via surface phase transformation, achieving excellent long‐term photoelectrochemical durability.

Additionally, Wang et al. [68] investigate the role of metal vacancies, specifically copper vacancies, in enhancing the performance of CuO‐based photocathodes for photoelectrochemical (PEC) water splitting. By systematically tuning the oxygen partial pressure during synthesis, the authors controlled the concentration of Cu vacancies within the CuO lattice. In this work, the authors controlled the formation of Cu vacancies as a passivating strategy achieved through intrinsic defect engineering, rather than through the application of an external overlayer. These vacancies were found to significantly improve the charge carrier density, as well as charge separation and transfer efficiency within the semiconductor. As a result, the CuO photocathode prepared under pure O_2_ exhibited a remarkable 100% increase in photocurrent density compared to samples fabricated in air, reaching values exceeding 1.8 mA cm^−2^. Importantly, this enhancement was achieved without compromising structural integrity or stability under operating conditions. The beneficial effect of Cu vacancies was also confirmed across other Cu‐based photocathodes, highlighting the generality of this defect‐engineering approach.

In the same direction, Qu et al. [69] reported the synthesis of nanoporous CuBi_2_O_4_ (np‐CBO) photocathodes and the deliberate modulation of their surface point defects through rapid thermal processing (RTP) under controlled atmospheres (O_2_, N_2_, and vacuum). The O_2_‐RTP treatment was found to markedly increase the charge carrier density and enhance both charge transport and transfer properties, while preserving the nanoporous morphology. Structural and spectroscopic analyses revealed that the O_2_ treatment induced the formation of copper vacancies while simultaneously reducing the concentration of Cu⁺ species and oxygen vacancies. This defect configuration effectively improved the electrochemically active surface area and facilitated efficient charge extraction, boosting the PEC performance and stability of bare CBO. As a result, the optimized np‐CBO–O_2_ photocathode exhibited an outstanding photocurrent density of −1.81 mA cm^−2^ at 0.4 V versus RHE under simulated sunlight. Furthermore, sequential RTP cycling between oxidizing and inert environments (O_2_–N_2_–O_2_–N_2_) demonstrated the reversibility of defect formation and the corresponding modulation of photocurrent response, confirming the dynamic control achievable through this approach.

**FIGURE 15:**
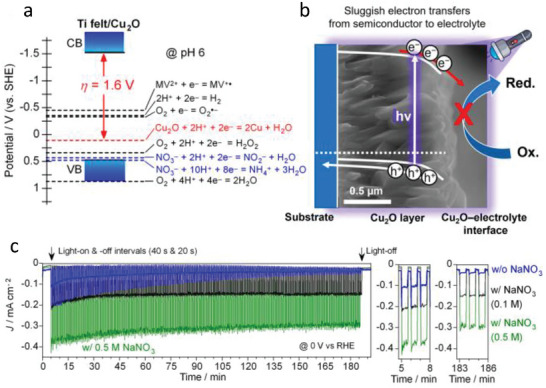
a) Energy band positions of Cu_2_O and PEC reduction reaction potentials of electron acceptors at pH 6.0. b) Scheme of electron transfers at the Cu_2_O semiconductor−electrolyte interface. c) Photoelectrostability test in 0.5 m Na_2_SO_4_ with and without NaNO_3_ (0.1 and 0.5 m) for 3 h under chopped light irradiation at 0.0 V_RHE_. Reproduced with permission from Surya et al. [70], Copyright 2025, ACS.

Recently, Surya and co‐workers systematically evaluated the photostability of Cu_2_O (using Ti felt as electrical contact in Cu_2_O photocathodes) across different reduction reactions [70]. In Cu_2_O under illumination, the sluggish electron transfer to solution‐phase acceptors promoted self‐reduction of Cu_2_O (Cu_2_O + 2H⁺ + 2e^−^ → 2Cu + H_2_O), leading to photocorrosion and diminished photocurrent responses. The authors revealed that nitrate reduction (NO_3_RR) significantly suppressed photocorrosion by providing a more favorable electron‐transfer pathway. This stabilization mechanism is best described as surface passivation by adsorbates, in which nitrate species adsorb onto the Cu‐based photocathode, mitigating surface degradation during operation. During NO_3_RR, the photocurrent remained stable at 0.0 V versus RHE under blue LED irradiation (λ = 456 nm), with minimal degradation observed over extended operation (Figure 15a,b). Faradaic efficiencies of approximately 81% for nitrite and 20% for ammonia were obtained, indicating selective conversion while maintaining electrode integrity. Structural and morphological analyses confirmed that nitrate adsorption on the Cu_2_O surface effectively redirected photogenerated electrons away from self‐reduction, thereby mitigating photocorrosion. Langmuir adsorption isotherms further supported this mechanism, showing preferential binding of NO_3_^−^ over competing anions (e.g., SO_4_^2−^). This adsorption‐driven stabilization facilitated the continuous reduction of *NO_3_ to *NO_2_, while regenerating active sites for further NO_3_^−^ adsorption, thereby maintaining activity and durability. Substrate effects were also examined by comparing Cu_2_O deposited on titanium felt and FTO. The Ti felt/Cu_2_O photocathode displayed significantly enhanced photocurrent densities relative to FTO/Cu_2_O, attributable to the 3D fiber architecture that increased both Cu_2_O–electrolyte and Cu_2_O–substrate interfacial areas. Importantly, Cu_2_O photocathodes retained their stability during NO_3_RR, as confirmed by extended PEC testing (Figure 15c). In contrast, substantial photocorrosion was observed during oxygen reduction and methyl viologen reduction, despite their nominally favorable reaction kinetics, underscoring the unique stabilizing role of nitrate adsorption.

The choice of passivation strategy must be tailored to the specific Cu‐based material and its dominant surface defects. Importantly, passivation layers can often be combined synergistically with protective and catalytic layers, offering multifunctional benefits. However, these layers often require fine‐tuning of thickness, composition, or treatment conditions, which can complicate fabrication and reduce throughput for industrial applications. When carefully engineered, surface passivation plays a crucial role in unlocking the full photoelectrochemical potential of Cu‐based photocathodes by extending carrier lifetimes and stabilizing interfacial charge transfer processes.

To avoid possible ambiguities, we would like to clarify and summarize the conceptual framework used in this review to distinguish among electron‐selective layers (ESLs), protective layers, and surface passivation strategies. In our framework, ESLs are defined primarily by their electronic function: they exhibit appropriate band alignment with the Cu‐based absorber, thereby promoting efficient extraction of photogenerated electrons and suppressing interfacial recombination. Protective layers, in contrast, are conceived as predominantly physical barriers that shield the semiconductor from photocorrosion by limiting direct contact between the absorber and the electrolyte; their stabilizing role does not necessarily require favorable band energetics. Surface passivation strategies constitute a third, distinct category, characterized by their ability to actively modify the surface chemistry, typically by passivating defects or suppressing surface states, to enhance both stability and charge‐transfer properties. Although these categories highlight different dominant mechanisms, they are not exclusive. Indeed, some materials frequently used in Cu‐based photocathodes, such as TiO_2_ or ZnO, may simultaneously fulfill more than one of these roles, acting for example as both an electron‐selective and a protective layer.

### 3.6 Use of Cocatalysts

The integration of cocatalysts onto Cu‐based photocathodes is a widely adopted strategy to enhance the kinetics of surface reactions and stabilize the semiconductor–electrolyte interface. In particular for HER, sluggish interfacial kinetics and competing recombination pathways can significantly limit the efficiency of charge utilization. Co‐catalysts facilitate faster interfacial electron transfer, reduce overpotentials, and often act as a physical buffer that protects the photocathode surface from direct interaction with the electrolyte, thereby mitigating photocorrosion.

A broad range of inorganic cocatalysts has been explored for this purpose. Noble metals such as gold (Au), silver (Ag) [71], platinum (Pt) and ruthenium (Ru) remain among the most effective HER catalysts, offering near‐zero overpotential and fast charge transfer rates. In this direction, Chen and co‐workers [72] designed ternary Au/Cu_2_O/Pt photoelectrodes to enhance light harvesting and charge separation for PEC water splitting. By spatially depositing ultrathin Au layers beneath Cu_2_O nanogranules and Pt cocatalyst layers on their surface, they combined the plasmonic sensitization effect of Au with the electron collection capability of Pt. The Au layer acted as a plasmonic photosensitizer, intensifying visible light absorption and generating hot electrons via surface plasmon resonance, while the Pt co‐catalytic layer facilitated efficient extraction and transfer of photoinduced electrons. This synergistic configuration was validated through IPCE, EIS, and SPV analyses, and delivered a photocurrent density of −3.55 mA cm^−2^ at 0 V versus RHE, representing a 4.6‐fold improvement over bare Cu_2_O. The authors were able to obtained stable photocurrent densities over 4000s, compared with a minimized 59% in photocurrent in the same period in bare Cu_2_O. ‐

However, due to cost and scarcity concerns, significant effort has been devoted to developing earth‐abundant alternatives, including nickel–iron (Ni‐Fe), cobalt phosphide (Co‐P), and molybdenum disulfide (MoS_2_), among others. These materials not only demonstrate promising HER activity but also exhibit good adhesion to oxide surfaces and chemical stability under reductive conditions. In this direction, Qi et al. [73] demonstrated that electrodeposition of ultrathin NiFe‐layered double hydroxide (NiFe‐LDH) overlayers onto Cu_2_O photocathodes significantly enhances their performance for PEC water splitting. The NiFe‐LDH coating provided an appropriate band alignment that facilitated charge transfer and reduced surface resistance, leading to a seven‐fold increase in photocurrent density (0.49 mA cm^−2^ at −0.25 V vs Ag/AgCl compared to 0.07 mA cm^−2^ for bare Cu_2_O). Electrochemical impedance spectroscopy confirmed that the NiFe‐LDH layers lowered charge‐transfer resistance, enabling efficient electron injection into the electrolyte. Importantly, these modified photocathodes delivered both high photocurrents and long‐term stability under mild operating conditions, sustaining 78% Faradaic efficiency for hydrogen evolution after 8 h of continuous operation at −0.2 V versus Ag/AgCl. The study also provided critical insights into the stability of Cu_2_O under illumination. Bare Cu_2_O is notoriously unstable because photogenerated electrons reduce it to metallic Cu while holes oxidize it to CuO, rapidly degrading the electrode surface. Conventional testing conditions, typically around 0 V versus RHE, favor this reduction pathway and explain the poor stability generally reported. However, the authors showed that operating Cu_2_O/NiFe‐LDH electrodes at −0.2 V versus Ag/AgCl and pH 6.5 places the material within its intrinsic stability window, allowing continuous operation for over 40 h without photocurrent loss. Additional experiments at more negative biases (−0.6 V vs Ag/AgCl) revealed that NiFe‐LDH coatings can further extend stability even under harsher conditions, though the primary aim of this work was to optimize Cu_2_O within its natural stability regime rather than under extreme bias. These findings underscore the dual role of NiFe‐LDH, enhancing charge transfer while enabling durable Cu_2_O operation under low‐bias or self‐biased PEC conditions.

On the other hand, Burungale and co‐workers fabricated a cobalt‐phosphate (Co‐Pi) decorated TiO_2_–Cu_2_O heterojunction photoanode through hydrothermal and electrodeposition methods [74]. TiO_2_ was first deposited on FTO substrates via hydrothermal synthesis, followed by electrodeposition of a Cu_2_O layer. Subsequently, the surface was further modified with a thin Co‐Pi overlayer using photo‐assisted electrodeposition. Structural and optical characterization by X‐ray diffraction, FESEM, XPS, and UV–Vis spectroscopy confirmed the successful formation of the heterostructure. Electrochemical measurements demonstrated a substantial enhancement in PEC performance. The deposition of Cu_2_O increased the photocurrent density of bare TiO_2_ from 1.13 to 1.66 mA cm^−2^ at 1.23 V versus RHE, while the introduction of Co‐Pi further boosted the photocurrent to 1.94 mA cm^−2^ at the same potential. These improvements were attributed to the synergistic effects of the TiO_2_/Cu_2_O p–n heterojunction, which enhanced visible‐light absorption and promoted efficient charge separation, along with the Co‐Pi layer that facilitated hole extraction at the electrode–electrolyte interface. A critical advantage of the Co‐Pi decorated TiO_2_–Cu_2_O photoanode was its improved stability under illumination. Pure Cu_2_O electrodes exhibited rapid photocurrent decay, with a decrease of nearly 84% of their initial value within 600 s, primarily due to self‐oxidation of Cu_2_O into CuO caused by accumulated photogenerated holes. In contrast, the TiO_2_–Cu_2_O–CoPi photoanode showed only a 34% decrease over the same period. This enhanced stability was explained by the type‐II band alignment between TiO_2_ and Cu_2_O, which promoted spatial charge separation, and by the Co‐Pi co‐catalyst, which selectively scavenged holes from the Cu_2_O surface. Together, these features minimized hole accumulation, thereby suppressing photocorrosion and enabling more durable PEC operation. This result highlights the dual role of the heterojunction and the co‐catalyst in simultaneously enhancing performance and prolonging electrode lifetime.

**FIGURE 16:**
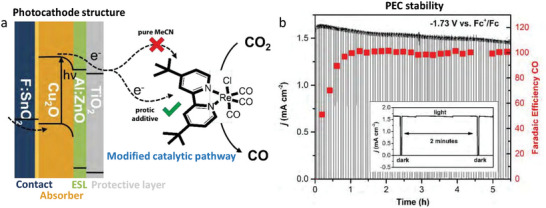
a) Scheme of the PEC CO_2_ reduction process involving protected Cu_2_O photocathodes and a Re‐based molecular catalyst. b) Chronoamperometry and CO evolution efficiency of the Cu_2_O photocathode under chopped light at a constant potential of ‐1.73 V versus Fc^+^ /Fc with 2 mm Re(tBu‐bipy)(CO)_3_Cl and 7.5 m MeOH under CO_2_. Both current density (black line) and faradaic efficiency towards carbon monoxide (red dots) are reported. The inset shows a zoomed view of the recorded current, showing the absence of dark current and the occasional changes in current density due to CO gas bubbles detaching from the electrode surface. Only little performance loss is observed over 5.5 h and can be mainly attributed to increased light absorption by the catalyst due to evaporation of the solvent. Reproduced and adapted with permission from Schreier et al. [75], Copyright 2015, RSC.

Beyond solid‐state catalysts, molecular co‐catalysts, such as cobalt‐based complexes or organometallic hydrogen evolution catalysts, have also been employed, particularly in hybrid systems. These can offer high activity and tunable functionality, though their long‐term stability remains a challenge. In their pioneering work, back in 2014, Schreier et al. [75] the authors demonstrated the efficient photoelectrochemical reduction of CO_2_ to CO on Cu_2_O photocathodes protected with TiO_2_ and functionalized with a rhenium‐based molecular catalyst (Figure 16a). The system delivered photovoltages up to 560 mV and photocurrent densities of 2.1 mA cm^−2^, representing the highest reported photocurrent for CO_2_ reduction on an oxide semiconductor. TiO_2_ protection ensured stable and selective operation for several hours, while mechanistic studies revealed an unexpected charge‐transfer limitation at the TiO_2_/catalyst interface. This barrier was overcome by introducing protic electrolyte additives, which modified the catalytic pathway to bypass charged intermediates. The findings not only highlight a viable strategy for stabilizing Cu_2_O toward CO_2_ reduction but also provide broadly applicable insights into charge‐transfer dynamics in semiconductor–molecular catalyst assemblies protected by oxide overlayers. The long‐term stability (Figure 16b) of the protected Cu_2_O photocathodes was evaluated under CO_2_ reduction conditions by introducing protic additives, which enabled efficient catalytic turnover. At −1.73 V versus Fc⁺/Fc, close to the onset of the first catalyst reduction on a glassy carbon electrode, the system achieved sustained photocurrent and quantitative CO production over several hours of operation under CO_2_‐saturated electrolyte and chopped illumination. Stability tests revealed negligible dark current throughout more than 5 h, confirming that the observed current originated from true photoactivity rather than photocorrosion, an effect further ensured by the protective TiO_2_ overlayer. The periodic photocurrent perturbations were attributed to the detachment of CO bubbles from the electrode surface, consistent with gas chromatography analyses that confirmed selective CO generation with only trace H_2_ production. The current yield was fully quantitative within experimental error, and only a minor decrease in photocurrent after 5.5 h was observed, attributed to solvent evaporation and increased catalyst concentration. Remarkably, this represents the longest reported stability for an oxide‐based photocathode toward CO_2_ reduction, and the ability to operate at potentials where silicon photocathodes fail to exhibit catalytic currents highlights the synergistic contribution of device photovoltage and the modified catalytic pathway induced by protic additives.

Recently, Yu et al. [76] addressed the severe photocorrosion and rapid charge recombination that typically limit the application of Cu_2_O in PEC water splitting by introducing a novel surface modification strategy. A Ni‐doped metal–organic framework, Cu_3_(BTC)_2_ (Ni‐CuBTC), was in situ constructed on electrodeposited Cu_2_O via solvothermal synthesis followed by ion exchange. Acting simultaneously as a photosensitizer, protective layer, and co‐catalyst, the Ni‐CuBTC decoration extended the light absorption edge to ≈800 nm, positively shifted the flat band potential to 0.4 V, and reduced the Tafel slope to 74 mV dec^−1^, thereby enhancing charge separation and transfer while accelerating HER kinetics. As a result, the modified Cu_2_O/Ni‐CuBTC photocathodes delivered a photocurrent density of −1.51 mA cm^−2^ at 0 V versus RHE under AM 1.5 G illumination, representing a 3.4‐fold improvement compared to pristine Cu_2_O. Beyond performance gains, the incorporation of Ni‐CuBTC significantly improved the operational durability of Cu_2_O photocathodes. While bare Cu_2_O typically suffers from rapid degradation due to self‐oxidation and photocorrosion, the decorated photocathodes retained 54.7% of their photocurrent at 0.5 V versus RHE after 1200 s of continuous illumination. This enhancement highlights the protective role of the Ni‐CuBTC overlayer, which not only prevents excessive hole accumulation on the Cu_2_O surface but also promotes efficient carrier extraction, thereby mitigating degradation pathways. These findings establish Ni‐CuBTC decoration as a low‐cost and effective strategy to stabilize Cu_2_O photocathodes, positioning metal–organic frameworks as promising candidates for durable PEC water‐splitting applications.

In light of the examples discussed above, co‐catalysts not only facilitate charge separation by spatially isolating the reaction site from the absorber, thereby mitigating local photodegradation and surface recombination, but also provide synergistic benefits when combined with electron‐extraction or protective layers. Together, these effects enhance both photocurrent and stability, underscoring the pivotal role of cocatalysts in designing durable, high‐performance Cu‐based photocathodes. However, many high‐performance catalysts are noble metals, which limit cost‐effectiveness and large‐scale deployment. Identifying earth‐abundant alternatives remains an ongoing challenge.

### 3.7 Synergistic Strategies

While individual stabilization strategies have shown measurable improvements in the performance and durability of Cu‐based photocathodes, the most promising advances have emerged from the integration of multiple complementary approaches. These synergistic strategies combine interfacial engineering, surface passivation strategies, protective coatings, and co‐catalyst integration into multi‐layered or hybrid architectures, enabling enhanced control over charge dynamics, chemical stability, and interfacial selectivity. For instance, pairing a TiO_2_ electron‐extraction overlayer with a conformal ALD‐grown protective coating and a surface‐bound cocatalyst (such as Pt or MoS_2_) can lead to simultaneous improvements in charge separation efficiency, corrosion resistance, and reaction kinetics. Similarly, combining a hole‐selective underlayer (e.g., NiO_x_ or CuSCN) with surface passivation treatments and a highly conductive overlayer can mitigate both bulk and interfacial recombination while enhancing carrier extraction. These integrated systems allow for fine‐tuning of energetic band alignment, minimization of recombination losses, and protection of vulnerable interfaces from electrochemical degradation.

Pan et al. [77] shown designed a coaxial nanowire architecture integrating a buried Cu_2_O/Ga_2_O_3_ p–n junction, which enabled efficient light harvesting throughout the visible spectrum up to approximately 600 nm. This configuration yielded an impressive photocurrent density of ≈10 mA cm^−2^ at 0 V versus the reversible hydrogen electrode (RHE) and an onset potential of +1.0 V versus RHE. To mitigate the intrinsic photocorrosion of Cu_2_O, a conformal TiO_2_ protection layer was deposited via atomic layer deposition (ALD), providing both chemical and photostability without hindering charge transfer. This protective coating enabled continuous and stable operation for over 100 h under simulated solar illumination, marking a significant improvement compared to conventional Cu_2_O‐based systems. The incorporation of an Earth‐abundant NiMo catalyst further enhanced hydrogen evolution kinetics while maintaining structural integrity under alkaline conditions. The long‐term performance evaluation revealed that the photocurrent density retained approximately 90% of its initial value after 8 h of continuous operation at +0.5 V versus RHE, confirming the robust stability of the photocathode. The minor photocurrent decay was attributed primarily to electrolyte permeation through the amorphous TiO_2_ layer rather than to degradation of the semiconductor or catalyst. Importantly, the study highlighted the potential of interface engineering and optimized protection strategies to suppress photocorrosion and prolong device lifetime.

**FIGURE 17:**
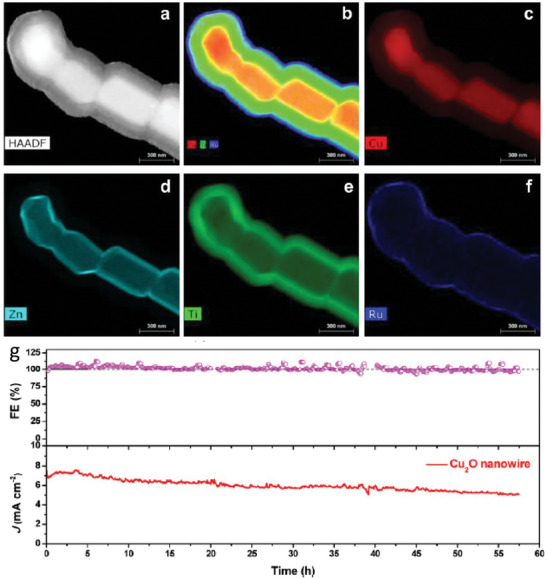
TEM and EDX characterization of the Cu_2_O NW with overlayers. a) HAADF image. b) Combined elemental mapping image. c–f) Element mapping of Cu, Zn, Ti, and Ru, respectively. g) Measured photocurrent and Faradaic efficiency for hydrogen evolution of Cu_2_O NW device under constant bias at 0 V versus RHE and simulated AM 1.5G illumination performed in pH = 5 electrolyte. Reproduced and adapted with permission from Luo et al. [78], Copyright 2016, ACS.

Also, Luo et al. [78] reported a significant advance in the design and fabrication of Cu_2_O nanowire (NW) photocathodes for solar‐driven hydrogen generation. Leveraging the abundance, low toxicity, and favorable electronic structure of Cu_2_O, the authors developed a novel synthetic route to grow vertically aligned Cu_2_O nanowire arrays on fluorine‐doped tin oxide (FTO) substrates with excellent phase purity and crystallinity. Compared with conventional planar Cu_2_O electrodes, the nanowire architecture effectively overcomes the mismatch between light absorption (axial direction) and charge‐carrier diffusion lengths (radial direction), enabling superior charge separation and collection efficiencies. The introduction of a conformal nanoscale p–n junction, an innovative blocking layer, and a durable protective overlayer resulted in a highly efficient and stable Cu_2_O NW/AZO/TiO_2_/RuO_x_ photocathode (Figure 17a–f) capable of achieving photocurrent densities up to 10 mA cm^−2^ at 0 V versus RHE, approaching the theoretical limit for this material. This performance establishes a new benchmark for oxide‐based photoelectrodes in photoelectrochemical (PEC) water splitting. Particular attention was given to assessing the long‐term operational stability of the nanowire photocathodes. Under continuous AM 1.5G illumination and an applied potential of 0 V versus RHE, the Cu_2_O NW/AZO/TiO_2_/RuO_x_ system exhibited outstanding stability over 55 h, maintaining a nearly constant photocurrent and a Faradaic efficiency close to 100% for hydrogen evolution (Figure 17g). Remarkably, post‐test analyses revealed no observable structural degradation, pinhole formation, or surface damage, confirming the robustness of both the Cu_2_O nanostructure and the protective overlayer in mildly acidic aqueous media (pH = 5). Furthermore, upon electrolyte replacement, the photocathode's performance nearly recovered to its initial level, indicating that minor current loss likely originated from electrolyte decomposition rather than photocorrosion of the semiconductor itself.

**FIGURE 18:**
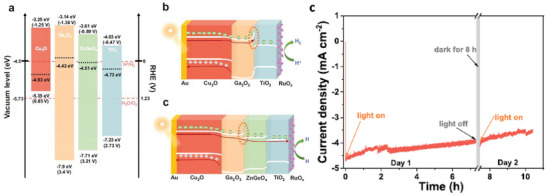
Energy diagrams and extracted resistance. a) Energy diagrams of Cu_2_O, Ga_2_O_3_, ZnGeO_x_, and TiO_2_ (from left to right) derived from Kelvin probe force microscopy (KPFM), XPS valence‐band spectra and the Tauc plots. b) Schematic diagram of the photogenerated carrier transport behavior in the Cu_2_O/Ga_2_O_3_/TiO_2_/ RuO_x_ photocathode under illumination. c) The stability measurement of the Cu_2_O/Ga_2_O_3_/ZnGeO_x_/TiO_2_ photocathode at 0 V versus RHE under simulated 1 Sun AM 1.5 G. All measurements were performed in pH = 5 phosphate–sulfate electrolyte. Reproduced and adapted with permission from Cheng et al. [79], Copyright 2023, Springer Nature.

In other interesting work, Cheng et al. [79] addressed the challenge of increasing the photovoltage of Cu_2_O photocathodes, a critical factor for achieving efficient PEC water splitting. They compared single‐buffer (Ga_2_O_3_) and dual‐buffer (Ga_2_O_3_/ZnGeO_x_) architectures, demonstrating that the introduction of ZnGeO_x_ created a favorable energy‐level gradient between Ga_2_O_3_ and TiO_2_ (Figure 18a,b). This modification eliminated interfacial barriers, leading to a 0.16 V increase in onset potential and raising the photovoltage to 1.07 V. Operando electrochemical impedance spectroscopy confirmed improved charge transfer dynamics arising from the dual‐layer design. While this work highlighted the potential of buffer‐layer engineering to boost Cu_2_O photovoltage, it also underscored remaining limitations, such as the theoretical gap to the 1.6 V maximum, the high resistance of Ga_2_O_3_, and the detrimental role of interfacial defects and surface states (e.g., Cu⁰, Cu^2^⁺) in recombination. The authors proposed that strategies such as heteroatom doping of Ga_2_O_3_, defect control, and development of high‐quality single‐crystal Cu_2_O could further advance the efficiency of these photocathodes. As shown in Figure 18c, the study established a dual‐buffer engineering as an effective route to enhance photovoltage while pointing to key directions for continued optimization.

Prévot and co‐workers [8], were able to prepare delafossite CuFeO_2_ photocathodes on transparent conductive substrates via a straightforward sol–gel citrate–nitrate method, enabling precise thickness control and oxygen intercalation. These strategies improved majority carrier density and facilitated photogenerated charge extraction, achieving sacrificial photocurrents of up to 1.51 mA cm^−2^ at +0.35 V versus RHE under 1‐sun illumination. Despite the intrinsic limitations in hydrogen evolution catalysis, electrochemical impedance spectroscopy revealed that electrons could be efficiently extracted from the conduction band before recombination into mid‐gap trap states. By applying oxide overlayers and platinum cocatalysts, sustained photocurrents for solar hydrogen production were realized, with values of 0.4 mA cm^−2^ at 0 V versus RHE and 0.8 mA cm^−2^ at –0.2 V versus RHE, supported by 100% faradaic efficiency for H_2_ evolution. Unlike many photocathodes prone to rapid degradation, bare CuFeO_2_ demonstrated exceptional operational stability. Chronoamperometric tests at +0.4 V versus RHE under chopped illumination showed no photocurrent loss over 40 h in oxygen‐saturated electrolytes, with only a minor ≈10% decrease during the initial hours. Structural analyses (Raman and XRD) confirmed the chemical integrity of the electrode after prolonged PEC operation. Similarly, cyclic voltammetry over the potential window of +0.3 to +0.9 V versus RHE revealed no signs of degradation, establishing CuFeO_2_ as one of the few oxide photocathodes intrinsically stable in alkaline electrolytes without protective coatings. However, at potentials more negative than +0.1 V versus RHE, evidence of copper and iron reduction emerged, highlighting the potential‐dependent limits of stability. Furthermore, the incorporation of AZO/TiO_2_ overlayers via ALD and Pt cocatalyst deposition significantly boosted photocurrent while maintaining stability. Under chopped illumination, the CuFeO_2_/AZO/TiO_2_/Pt photocathode sustained stable currents for at least 50 min, with faradaic hydrogen generation verified quantitatively. Although this architecture introduced a cathodic shift in the photocurrent onset (+0.4 V vs RHE), it demonstrated the efficacy of combining surface engineering with cocatalysts to simultaneously enhance PEC performance and durability.

On the other hand, Wei et al. [80] developed a stabilized CuBi_2_O_4_ (CBO)‐based photocathode by integrating ZnSe (ZS) and P25 layers to address the intrinsic limitations of poor stability and severe charge recombination in CBO. The resulting CBO/ZS/P25 photocathode exhibited a photocurrent density of 0.43 mA cm^−2^, more than double that of pristine CBO. Mechanistic analysis revealed that the high performance stemmed from the heterojunction between ZS and P25, which facilitated charge separation, the high electron mobility of ZS that accelerated electron transfer, and the hole extraction capacity of CBO. The P25 overlayer also served as a protective coating, reducing direct contact with the electrolyte and contributing to the overall durability of the composite photocathode. A key feature of the CBO/ZS/P25 system was its remarkable photostability compared to pristine CBO and CBO/ZS. While bare CBO photocathodes lost nearly 60% of their photocurrent within 5000 s of operation, and the CBO/ZS composite also showed clear degradation, the addition of the P25 protective layer preserved ≈90% of the photocurrent after 5000 s under continuous illumination. This marked improvement indicates that P25 effectively suppressed photocorrosion and prolonged the operational lifetime of the photocathode, outperforming other CBO‐based composites prepared under similar conditions. Although the absolute PEC activity remains moderate, the significant enhancement in both performance and stability, achieved without precious metals or expensive cocatalysts, demonstrates the promise of this low‐cost, green strategy for advancing CBO‐based photocathodes toward sustainable solar hydrogen production.

Azevedo et al. [81] reported a novel method to tackle the major challenge with Cu_2_O, which lies in its limited chemical stability in aqueous environments. To address this, a simple and low‐cost steam treatment has been reported to yield highly stable Cu_2_O‐based photocathodes for H_2_ production. Applied to Cu_2_O/AZO/TiO_2_ multilayer structures, this strategy enhanced durability when combined with either RuOx or Pt co‐catalysts. The optimized electrodes achieved photocurrents exceeding ≈5 mA cm^−2^ with 90% retention over more than 50 h of chopped illumination (biased at 0 V RHE in pH 5 electrolyte). Beyond these record operational stabilities, the study also underscored the crucial influence of surface morphological transformations induced by the treatment in enabling long‐term photocathode stability.

Additionally, Burns et al. [82] advanced Cu‐based photocathode design by developing CuO|CuBi_2_O_4_ heterojunctions stabilized with protective overlayers of TiO_2_, NiO, or MgO and employing Pt or MoS_2_ nanoparticles as co‐catalysts for hydrogen evolution. Among the materials tested, MgO was highlighted as a novel passivation layer for photocathodes, showing superior performance in enhancing stability and charge transfer. The optimized CuO|CuBi_2_O_4_|MgO|Pt configuration achieved stable photocurrents of approximately −0.2 mA cm^−2^ for over 3 h in neutral water with Faradaic efficiencies near 90%. Tandem, bias‐free water splitting was also demonstrated by pairing this photocathode with a dye‐sensitized TiO_2_ photoanode, sustaining photocurrents of 75 µA cm^−2^ for 1 h with high hydrogen yields. Comprehensive spectroelectrochemical and transient absorption studies revealed that protective coatings suppressed surface recombination, slowed degradation processes, and stabilized charge transfer pathways, with MgO proving the most effective among the tested oxides. While challenges such as pinhole formation in the protective layers remain, this work provided critical mechanistic insights into recombination and stabilization strategies for Cu‐based photocathodes and established abundant, low‐cost oxides as viable protective materials for scalable tandem PEC water‐splitting systems.

**FIGURE 19:**
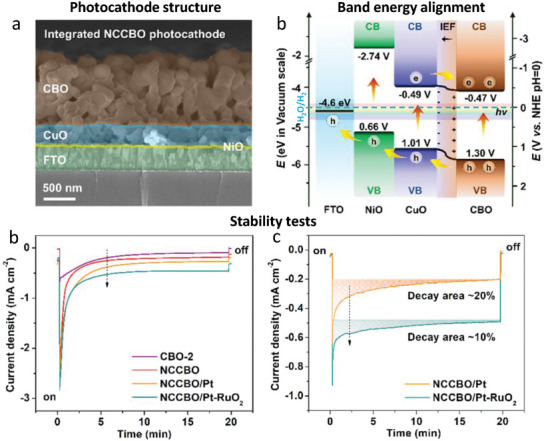
a) Cross‐section SEM image and b) band energy diagram of the integrated NCCBO photocathode. c) Stability tests of the photocathodes at 0.4 V versus RHE. d) Stability tests 0.7 and 0.8 V versus RHE for NCCBO/Pt and NCCBO/Pt‐RuO_2_, respectively. Reproduced and adapted with permission from Wang et al. [83], Copyright 2025, Wiley.

Very recently, Wang and co‐workers reported a highly efficient porous CuBi_2_O_4_ (p‐CBO) photocathodes were developed through a synergistic surface and interface engineering strategy, integrating CuO/CBO heterojunctions with NiO hole‐transport layers with adequate band‐alignment (Figure 19a,b) [83]. This architecture significantly enhanced bulk charge separation and interfacial transport, enabling record‐high PEC performance. After surface platinization, the photocathode reached a photocurrent density of 3.50 mA cm^−2^ at 0.40 V versus RHE with an applied bias photon‐to‐current efficiency (ABPE) of 1.60%. When coupled with an oxygen‐deficient TiO_2_₋_x_ photoanode, the integrated device achieved a solar‐to‐hydrogen (STH) efficiency of 0.31%, establishing a new benchmark for CBO‐based tandem systems. Despite the improvements in charge separation and surface kinetics, unmodified NCCBO photocathodes still suffered from rapid photocurrent decay due to the intrinsic instability of the CuO layer, which is highly prone to photocathodic corrosion. In fact, residual photocurrent densities of bare CuO electrodes dropped to ≈10 µA cm^−2^ within 20 min, far below the values observed for CBO controls. Remarkably, the incorporation of surface HER co‐catalysts provided substantial stabilization. Among them, Pt‐RuO_2_‐modified NCCBO demonstrated superior durability compared to Pt‐only modified samples, even under more negative applied potentials (0.70–0.80 V versus RHE). Post‐PEC XPS analyses revealed that Pt‐RuO_2_ effectively suppressed the reduction of Cu^2^⁺ to Cu⁺, thereby mitigating photocorrosion (Figure 19c,d). This stabilization was attributed to the accelerated HER kinetics and reduced electron accumulation at the surface enabled by the dual co‐catalyst.

Also, in the recent work from Chen et al. [84], the authors reported an efficient ternary Cu_2_O/Cu/CeO_2_ heterojunction photocathode was designed and integrated into a plasma‐coupled PEC system for nitrogen reduction. The incorporation of CeO_2_ not only reduced the resistivity of the conductive substrate but also actively participated in the catalytic process, thereby enhancing overall photoelectrocatalytic activity. Under optimized plasma discharge conditions and an applied potential of −0.7 V versus RHE, the heterostructured photocathode demonstrated remarkable performance, achieving NO_2_^−^ and NO_3_^−^ concentrations of 899.53 and 107.36 ppm, respectively, alongside an ammonia production rate of 218.09 µmol h^−1^ cm^−2^. The ternary architecture facilitated a type‐II band alignment that promoted efficient charge separation and transfer, while metallic Cu served as an electron reservoir to drive nitrogen reduction. Meanwhile, the synergistic contributions of Cu_2_O and CeO_2_ enhanced water dissociation, supplying active *H intermediates that selectively hydrogenated NOx species to ammonia. Importantly, this configuration also addressed one of the fundamental challenges limiting Cu_2_O‐based photocathodes, which is their intrinsic susceptibility to photocorrosion. While Cu_2_O possesses favorable electronic properties for PEC nitrogen reduction, its poor stability has hindered practical deployment. The introduction of CeO_2_ effectively mitigated this limitation by passivating surface states, improving charge separation, and stabilizing the photocathode during operation. The synergy between plasma activation and heterojunction engineering therefore not only boosted ammonia synthesis efficiency but also extended electrode durability under reductive conditions.

**FIGURE 20:**
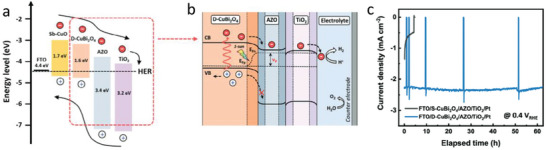
a) Scheme of the relative band energy positions in the photocathode. b) Characteristic band‐bending of p‐CuBi_2_O_4_ and n‐AZO/TiO_2_ in contact with an electrolyte under illumination. c) Long‐term PEC stability of S‐CuBi_2_O_4_ and D‐CuBi_2_O_4_ photocathodes at 0.4 V_RHE_ under illumination. Reproduced and adapted with permission from Choi et al. [47], Copyright 2025, Wiley.

In addition, in the previously mentioned study from Choi et al. [47], the authors improved the PEC performance of their D‐CuBi_2_O_4_ photocathodes by introducing n‐type Al‐doped ZnO (AZO) and TiO_2_ overlayers as electron transport layers, and Rh_2_P as an efficient cocatalyst for HER (Figure 20a,b). Under these optimized conditions, the resulting FTO/D‐CuBi_2_O_4_/AZO/TiO_2_/Rh_2_P photocathode delivered a photocurrent density of 2.56 mA cm^−2^ at 0 V versus RHE and an onset potential of 1.04 V versus RHE. Notably, it maintained outstanding operational stability for over 60 h of continuous photoelectrochemical operation (Figure 19c), underscoring its potential as a durable and efficient Cu‐based photocathode for solar hydrogen production.

Such multilayer designs have proven especially effective in long‐term operational tests, with several reports demonstrating stable photocurrent densities over 24 to 100 h of continuous illumination. The durability observed in these systems highlights the importance of addressing not just one, but multiple degradation pathways simultaneously, ranging from chemical corrosion to interfacial charge trapping and catalyst detachment.

As the field progresses, rationally designed architectures that exploit the synergy between functional layers are expected to play a central role in pushing Cu‐based photocathodes closer to practical deployment. A deeper understanding of interfacial interactions and degradation mechanisms will further enable the design of stable, efficient systems for solar‐driven hydrogen production and other photoelectrochemical reduction processes.

Combining multiple stabilization strategies represents the most promising approach to achieving highly efficient and durable Cu‐based photocathodes. However, this strategy is also the most expensive, as it often requires complex fabrication processes, costly materials (e.g., noble metals, ALD‐deposited oxides), and precise interface engineering. While synergistic designs excel in laboratory performance, their high cost and complexity pose significant challenges for scaling up to large‐area, practical devices.

## 4 Conclusions and Perspectives

The development of stable and efficient Cu‐based photocathodes for photoelectrochemical (PEC) reduction reactions has advanced considerably through a broad range of interfacial and surface engineering strategies. This review has discussed seven key approaches (optimization of the electrical contact with the substrate, use of hole‐selective layers, electron‐extraction overlayers, protective coatings, surface passivation strategies, co‐catalyst integration and synergistic strategies) all of which address distinct limitations related to charge transport, surface recombination, and chemical degradation.

**FIGURE 21:**
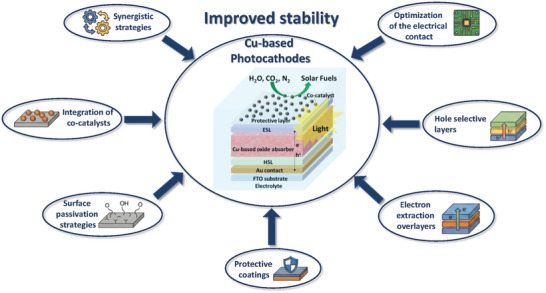
Scheme of the different analyzed strategies for improving the PEC stability in Cu‐based photocathodes revised in the present manuscript.

Individually, these strategies, summarized in Figure 21, have demonstrated significant performance gains. Metallic interlayers and hole‐selective underlayers improve back‐contact quality and facilitate hole extraction; overlayers and protective coatings enhance interfacial stability and charge selectivity; and co‐catalysts accelerate surface kinetics while simultaneously protecting the photoabsorber from corrosion. However, the most durable and high‐performing Cu‐based photocathodes typically emerge from synergistic combinations of these strategies. Multilayered designs that concurrently address electrical, chemical, and catalytic challenges are proving essential for achieving sustained PEC operation under realistic solar‐driven conditions.

Despite these encouraging advances, several challenges must still be overcome before Cu‐based photocathodes can attain commercial viability. Scalability remains a key issue, as some of the most effective stabilization methods, such as atomic layer deposition (ALD), noble metal catalysts, or complex heterostructures, are often costly and difficult to implement over large areas. Furthermore, maintaining high performance under practical operating conditions, including variable pH, fluctuating light intensities, and extended operation beyond 1000 h, remains a critical hurdle. Integrating these stabilization strategies into low‐temperature, solution‐processable fabrication routes compatible with flexible or lightweight substrates also represents a major technological bottleneck.

4.1

An emerging and complementary strategy to improve the durability of Cu‐based photocathodes involves the use of non‐aqueous or mixed aqueous–organic electrolytes. In conventional aqueous environments, Cu_2_O, CuO, and related oxides are highly susceptible to photocorrosion, disproportionation, and dissolution under reductive conditions. Replacing or partially substituting water with organic solvents such as acetonitrile, methanol, or ionic liquids can substantially suppress these degradation pathways by expanding the electrochemical stability window and modulating interfacial reactions.

Beyond improved stability, non‐aqueous electrolytes offer opportunities to tune reaction selectivity. For example, in CO_2_ reduction, aprotic or ionic liquid‐based media can effectively suppress the competing hydrogen evolution reaction, favoring carbon‐based product formation. Similarly, mixed aqueous–organic systems can minimize side reactions while maintaining sufficient ionic conductivity. Importantly, this electrolyte‐based approach is orthogonal yet synergistic with interfacial engineering strategies such as protective coatings or cocatalyst integration, allowing for simultaneous optimization of both the solid–liquid and solid–solid interfaces.

Nevertheless, several challenges remain for the widespread adoption of non‐aqueous electrolytes in PEC devices, including relatively low ionic conductivity, possible solvent degradation under illumination or bias, and issues of scalability, safety, and environmental impact. Future research should thus aim at developing green, stable, and conductive electrolyte formulations capable of extending photocathode lifetime without compromising efficiency or sustainability.

4.2

Looking forward, progress in this field will rely on the design of multifunctional materials and interfaces that merge stability, selectivity, and scalability. Promising directions include the development of earth‐abundant, self‐healing co‐catalysts; conductive passivation overlayers; and solution‐processable hole‐selective layers tailored for Cu‐based oxides. In parallel, advanced in situ and operando characterization techniques will be vital for elucidating the complex degradation mechanisms occurring under operation, enabling the rational design of more resilient systems.

Additionally, realizing practical Cu‐based photocathodes for solar‐to‐fuel conversion will require holistic device‐level integration, where light absorption, charge transport, and interfacial chemistry are co‐optimized. With continued interdisciplinary collaboration across materials science, electrochemistry, and device engineering, Cu‐based photocathodes hold strong promise for scalable and sustainable solar‐driven fuel production.

A final remark concerns the urgent need for standardized protocols to evaluate the long‐term stability of Cu‐based photocathodes. The field remains fragmented in terms of testing conditions, which severely complicates any meaningful comparison between reported stability values. Significant discrepancies arise from the use of three‐electrode configurations, often employing buffered electrolytes or sacrificial agents, versus two‐electrode, unassisted systems that provide a far more stringent assessment of operational durability. Similarly, the widespread use of chopped‐light chronoamperometry can mask degradation processes by allowing partial self‐healing during the dark intervals, whereas continuous illumination tests offer a more realistic measure of photochemical robustness. To move toward reliable benchmarking, it is essential that studies report detailed and standardized parameters, including electrolyte composition and pH, applied bias conditions, illumination intensity and spectrum, and post‐mortem structural and chemical analyses. Establishing such unified reporting practices will be pivotal for enabling fair comparisons across studies and for accelerating progress toward truly durable Cu‐based photocathodes suitable for practical solar‐fuel applications.

## Conflicts of Interest

The authors declare no conflicts of interest.

## Data Availability

The authors have nothing to report.
